# Genetic Diversity and Population Structure of *Trypanosoma brucei* in Uganda: Implications for the Epidemiology of Sleeping Sickness and Nagana

**DOI:** 10.1371/journal.pntd.0003353

**Published:** 2015-02-19

**Authors:** Richard Echodu, Mark Sistrom, Rosemary Bateta, Grace Murilla, Loyce Okedi, Serap Aksoy, Chineme Enyioha, John Enyaru, Elizabeth Opiyo, Wendy Gibson, Adalgisa Caccone

**Affiliations:** 1 Faculty of Science, Gulu University, Gulu, Uganda; 2 Department of Ecology and Evolutionary Biology, Yale University, New Haven, Connecticut, United States of America; 3 Trypanosomiasis Research Centre, Kenya Agricultural Research Institute, Kikuyu, Kenya; 4 National Agricultural Research Organisation, National Livestock Resources Research Institute, Tororo, Uganda; 5 Department of Epidemiology of Microbial Diseases, Yale School of Public Health, New Haven, Connecticut, United States of America; 6 School of Biological Sciences, Makerere University, Kampala, Uganda; 7 School of Biological Sciences, University of Bristol, Bristol, United Kingdom; Institute of Tropical Medicine, BELGIUM

## Abstract

**Background:**

While Human African Trypanosomiasis (HAT) is in decline on the continent of Africa, the disease still remains a major health problem in Uganda. There are recurrent sporadic outbreaks in the traditionally endemic areas in south-east Uganda, and continued spread to new unaffected areas in central Uganda. We evaluated the evolutionary dynamics underpinning the origin of new foci and the impact of host species on parasite genetic diversity in Uganda. We genotyped 269 *Trypanosoma brucei* isolates collected from different regions in Uganda and southwestern Kenya at 17 microsatellite loci, and checked for the presence of the SRA gene that confers human infectivity to *T. b. rhodesiense*.

**Results:**

Both Bayesian clustering methods and Discriminant Analysis of Principal Components partition *Trypanosoma brucei* isolates obtained from Uganda and southwestern Kenya into three distinct genetic clusters. Clusters 1 and 3 include isolates from central and southern Uganda, while cluster 2 contains mostly isolates from southwestern Kenya. These three clusters are not sorted by subspecies designation (*T. b. brucei* vs *T. b. rhodesiense*), host or date of collection. The analyses also show evidence of genetic admixture among the three genetic clusters and long-range dispersal, suggesting recent and possibly on-going gene flow between them.

**Conclusions:**

Our results show that the expansion of the disease to the new foci in central Uganda occurred from the northward spread of *T. b. rhodesiense* (*Tbr*). They also confirm the emergence of the human infective strains (*Tbr*) from non-infective *T. b. brucei* (*Tbb*) strains of different genetic backgrounds, and the importance of cattle as *Tbr* reservoir, as confounders that shape the epidemiology of sleeping sickness in the region.

## Author Summary

Human African Trypanosomiasis (HAT) is a major health problem in Uganda, as there are recurrent sporadic outbreaks of the disease in traditionally endemic areas in south-east Uganda, and continued spread to new unaffected areas in central Uganda. In this study, we evaluate the evolutionary dynamics underpinning the origin of new disease foci and the impact of host species on parasite genetic diversity in Uganda. We found three distinct genetic clusters of *T*. *brucei* in Uganda and southwestern Kenya. Clusters 1 and 3 include isolates from central and southern Uganda, while cluster 2 contains mostly isolates from southwestern Kenya. These three clusters are not sorted by subspecies designation (*T*. *b*. *brucei* vs *T*. *b*. *rhodesiense*), host or date of collection. Our results show expansion of the disease to new foci in central Uganda occurred from the northward spread of *T*. *b*. *rhodesiense*. They also confirm the emergence of the human infective strains from non-infective T. b. brucei strains of different genetic backgrounds, and the importance of cattle as Tbr reservoir, as confounders that shape the epidemiology of sleeping sickness in the region.

## Introduction

*Trypanosoma brucei* is a unicellular protozoan parasite, which causes human and animal trypanosomiasis in tropical Africa, transmitted by tsetse flies (*Glossina spp)*. *Trypanosoma brucei* consists of three subspecies: *T*. *b*. *brucei* (*Tbb*), *T*. *b*. *gambiense* (*Tbg*), and *T*. *b*. *rhodesiense* (*Tbr*) that are morphologically indistinguishable and classified according to host specificity, type of disease, and geographical distribution [1–3]. *Tbr* and *Tbg* cause the acute and chronic forms of Human African Trypanosomiasis (HAT), respectively. *Tbr* is restricted to certain regions of East Africa, while *Tbg* is more widespread in West and Central Africa. Both forms of HAT have an overlapping distribution with the non-human infective *Tbb*, which infects a wide range of wild and domestic animals across the tsetse belt of tropical Africa and is one of the causative organisms of African Animal Trypanosomiasis (AAT) or Nagana. Both *Tbr* and *Tbb* can co-occur in the same non-human hosts as well as in the tsetse vector. However, recombination is known to happen only in the salivary glands of the tsetse [4]. *Tbr* is not a reproductively isolated taxon but regarded as a host-range variant of *Tbb* [5–7]. A single gene encoding the Serum Resistance Associated (SRA) protein allows *Tbr* to survive in humans [8]. This gene possesses two main alleles across the *Tbr* distribution [6–7] The human serum resistance associated gene is ubiquitous and conserved in *Tbr* throughout East Africa [6]and could potentially be spread naturally by genetic exchange between *Tbr* and *Tbb* [9].

**Fig 1:**
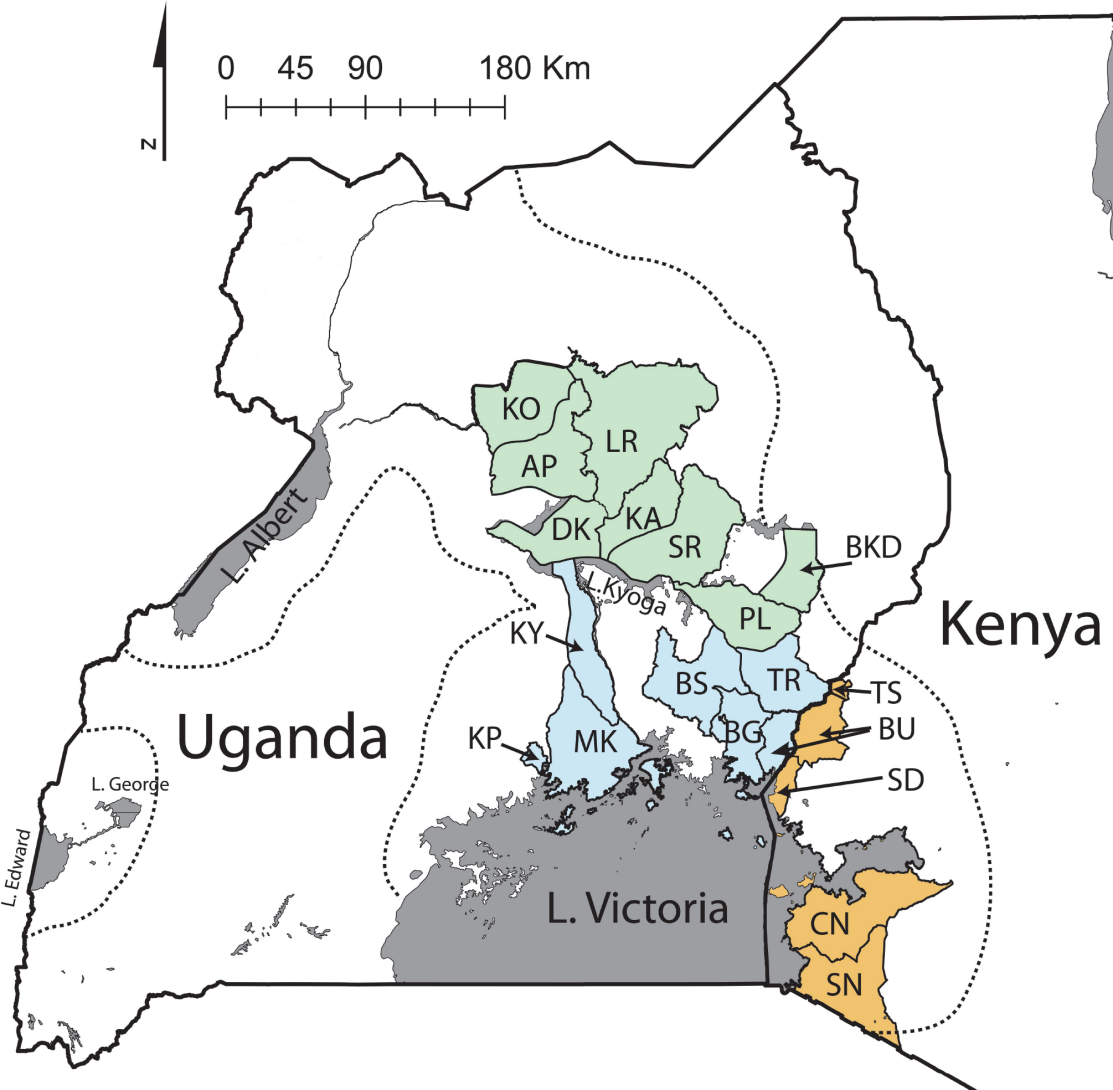
The 19 Ugandan and Kenyan districts from which *T*. *brucei* samples were collected. The dotted lines indicate the *G*. *f*. *fuscipes* distribution in the study region, and thus the distribution of *T*. *brucei*; there is a disjunct area of *G*. *f*. *fuscipes* around Lake George. Lakes (grey shading) are indicated by name. Districts are identified by two/three letter abbreviations (expanded in Table 1 and S1 Table). Districts are color-coded as follows: green—new foci of *T*. *b*. *rhodesiense* (*Tbr*) in central Uganda; blue—old foci of *Tbr* in southeastern Uganda; orange—foci of *Tbr* in western Kenya. The blue and green shaded areas separated by Lake Kyoga also demarcate the genetically distinct northern and southern *G*. *f*. *fuscipes* populations[16–7].

While HAT is in decline on the continent of Africa [10], the disease still remains a major health problem in Uganda, characterized by recurrent sporadic outbreaks in the traditional endemic areas and spread to new unaffected areas in central Uganda [11]. Uganda is currently the only country in sub-Saharan Africa known to harbor all three subspecies of *T*. *brucei*. The locations of districts affected by HAT are shown in Fig. 1 [11–15]. During most of the 20^th^ century, *Tbr* was limited to south-east Uganda in the old foci of Busoga (BS) and Bugiri (BG), and in areas bordering Tanzania and Kenya, such as Busia (BU), By the late 1980’s HAT appeared in Tororo (TR) and by 1998, HAT cases began to spread north and west being recorded in the Soroti (SR) district, north of Lake Kyoga in Central Uganda. From 2004 to date, all the districts in central Uganda—Kaberamaido (KA), Dokolo (DK), Lira (LR), Apac (AP), Kole (KO)—have reported HAT cases [15]. The affected areas increased in size from 13,820 to 34,843 km^2^, doubling the human population at risk [14]. *Tbr* and *Tbg* are now less than 120km apart. We refer to these foci in central Uganda as the new foci (Fig. 1). The epidemics in the new foci have been attributed to import of cattle carrying *Tbr* from disease endemic areas in the south [11], although recent work on the tsetse vector, *Glossina fuscipes fuscipes*, suggests that movement of susceptible flies from south to north could also be implicated in the emergence of disease in new foci [16–19]. Analyses of microsatellite and mitochondrial haplotype data show that the populations of *G*. *f*. *fuscipes* north and south of Lake Kyoga are genetically distinct and have identified long distance dispersal events [16, 17].

Population genetics studies have been carried out on *T*. *brucei* isolates across Africa, including HAT foci in Uganda and western Kenya. Analysis of *Tbr* isolates from the old foci in southeastern Uganda (BS, BG, BU, Fig. 1) by isoenzyme, RFLP, and microsatellite analyses show that they are relatively heterogeneous [20–25]. Genotype has been correlated with clinical presentation in patients and virulence in experimental mice [24]. Although it is assumed that *Tbr* spread from the old to the new foci, *Tbr* isolates from Soroti and Tororo (SR and TR respectively, Fig. 1) were genetically distinct from those in the old foci, but closely related to each other [25], which concurs with the idea that *Tbr* was introduced into Soroti via cattle from Tororo [10]. Microsatellite analysis (7 loci) of *Tbr* populations from Tororo/Soroti and Malawi showed that levels of genetic diversity were much higher in the Malawi focus, with evidence of recent genetic exchange between isolates [25]. The lack of genetic exchange and clonal, epidemic population structure of Tororo/Soroti *Tbr* agrees with the conclusions of previous population genetics studies [22, 23]. Thus, the local population structure of *Tbr* seems to depend on the relative amounts of clonal versus sexual reproduction, driven by transmission dynamics specific to the local conditions.

In this paper we used a set of 17 highly variable microsatellite loci [26–28] to investigate the patterns of genetic variation among 269 *Tbb* and *Tbr* isolates from Uganda and the neighbouring region of western Kenya in order to understand the extent of genetic exchange both within and between *Tbb* and *Tbr* and to investigate the origin and spread of HAT in Uganda. This is by far the most comprehensive study of genetic variation in Ugandan *T*. *brucei* yet undertaken. Understanding the population structure of *T*. *brucei* and the extent of genetic variation in both human infective and non-infective subspecies will reveal the potential for generation and spread of new human infective strains and is thus of critical relevance for disease control.

## Materials and Methods

### Trypanosomes and DNA purification

All 269 *T*. *brucei* isolate details are in Supplementary material (S1 Table). The *T*. *brucei* isolates were collected between 1959 and 2011 in 19 sites from the known parasite range in Uganda and western Kenya (Fig. 1). The isolates were obtained from various hosts (180 from humans, 57 from cattle, 1 from a sheep, 11 from pigs, 1 from a dog, 7 from wild animals and 12 from tsetse, S1 Table). Most of the samples (N = 194) were from archival cryopreserved collections, while 75 were collected in 2010 and 2011 mainly from Kole (KO) and Kaberamaido (KA). This is an important feature of this study, which aims to describe patterns of genetic variation and evolutionary processes of both *Tbb* and *Tbr* in all their potential hosts.

For these field samples, blood was collected on Whatman FTA (Fast Technology for Analysis of nucleic acids) cards (FTA is a registered trademark of GE Healthcare), which facilitates blood collection for nucleic DNA analysis. DNA extractions were carried out using DNeasy kits (Qiagen, Valencia, CA), following the manufacturers’ protocols. Other DNAs from isolates in the cryo collections were extracted by standard methods from cultured parasites (see S1 Table). For these isolates we chose material closest to the original field isolation to avoid selection bias through prolonged cell culture [29]. Trypanosome isolates from humans were collected for different studies according to local ethical guidelines and were treated anonymously.

### PCR test for taxonomic identification and microsatellite loci screen

All DNAs from the 2010 and 2011 field collections were screened using a diagnostic ITS based PCR test to separate *T*. *brucei* from other African trypanosomes [30]. All *T*. *brucei* samples were further tested for the presence of the *SRA* gene using the primer pairs SRA-R-SRA-F [31] and SRA H-SRA J [6]. Amplifications were carried out in a 25μl reaction volume containing 1X buffer (GoTaq colorless Promega), 1 mM each dNTP, 0.6 mM primers, 2 mM MgCl_2,_ 0.5 mg/ml BSA and 0.5 U Go Taq polymerase. The amplification involved a denaturation step at 95°C for 2 min, followed by 50 cycles each at 95°C for 35 s, 56°C for 35 s, 72°C for 1 min, with a final extension step at 72°C for 7 min. PCR products were visualized on 2% agarose gels.

Fluorescently labelled forward primers for seventeen *T*. *brucei* microsatellite loci were used for microsatellite genotyping. Their sequence and chromosomal locations are in S2 Table [26–28]. PCR amplifications were carried out using Type-it microsatellite PCR kit (Qiagen, Germany). 1μl of genomic DNA diluted to approximately 100ng/μl was amplified using 5μl of Type-it Master Mix and 1μl each of forward and reverse primers in a total reaction volume of 15μl. PCR reactions were carried out using an Eppendorf Mastercycler Pro thermocycler (Eppendorf, Germany) under the following PCR cycling profile: initialization step of 95°C for 4 minutes, followed by twelve touch-down cycles of 95°C for 30 seconds, 60–50°C for 25 seconds and 72°C for 30 seconds, an additional 30 cycles of 95°C for 30 seconds, 50°C for 25 seconds and 72°C for 30 seconds, and a final extension step of 72°C for 20 minutes. As template concentration for DNA samples extracted from FTA cards varied, genotyping of the field samples was repeated 2–5 times and genotype calls accepted only where replicates were concordant.

PCR products were multiplexed in groups of two or three before fragment analysis and sizing by capillary electrophoresis using an automatic 3730xl DNA Analyzer (Applied Biosystems Inc.). Allele sizes were determined using Genescan ROX-500 internal size standard for loci; TB1/8, TB5/2, TB6/7, TB9/6, TB10/5, TB11/13, Tryp51, Tryp67, Tryp55, Tryp53 and Tryp59 and Liz-500 internal size standard for loci; Tryp66, Tryp54, Tryp62, Tryp59 and Tryp53. In a 96-well microtitre plate, 1 μl of PCR product was added to 9 μl formamide and 0.5ul of either ROX500 or Liz500 size standard.

### Genetic diversity

Allele size calling was performed using GeneMarker version 2.4.0 (SoftGenetics, USA) and manually edited. Raw alleles were exported from GeneMarker to TANDEM version 1.0.9 [32] for allele binning. Genepop version 4.2 [33] was used to calculate number of alleles (N_a_), observed (H_o_) and expected (H_e_) heterozygosity levels under Hardy-Weinberg equilibrium (HWE) conditions. The same program was used to calculate allele richness (Ar; the number of alleles per locus, which is expected to be more sensitive to founder effects than is heterozygosity) and the inbreeding coefficient (Fis), one of the F statistics measuring genetic structure [34]. Fis measures the mean reduction in heterozygosity of an individual due to non-random mating in a population, thus the inbreeding within subpopulations, and ranges from -1 (all individuals heterozygotes) to +1 (no observed heterozygotes). Linkage disequilibrium (LD) was evaluated using the log likelihood ratio statistic (G—statistic) implemented in Genepop v4.2 [33].

### Population structure and differentiation

Using the Bayesian clustering method implemented in STRUCTURE version 2.3.3 [35], patterns of population structure, individual assignment to

sampling localities, and levels of genetic admixture were tested by identifying genetic

clusters without using a priori sampling information on the number of genetic groups in the data set. Bayesian clustering implemented in STRUCTURE v2.3.3 [35] was used to assign isolates to genetic clusters (K) according to the allele frequencies at each locus. Five independent runs for K = 1–10 were carried out. For all runs, an admixture model and independent allele frequencies were used with a burn-in value of 250,000 steps followed by 1,000,000 iterations. The optimal value of K was determined using STRUCTURE HARVESTER v0.6 [36] to calculate the ad hoc statistic “ΔK” [37]. Assignment of individual strains to a given cluster and levels of genetic admixture within each individual were assessed using STRUCTURE membership coefficients (*Q*-values), which represent the fraction of the sampled genome that has ancestry in a given cluster.

Genetic clustering between *T*. *brucei* isolates was also determined using Discriminant Analysis of Principal Components (DAPC) implemented in the R [38] package Adegenet [39]. This method is not model based as the previous one, and thus does not make assumptions on HWE or LD. It also tends to perform better when hierarchical and clinal structure is present [40]. DAPC comprises two steps: 1) a principal component step, where the dimensionality of the multilocus allelic data is reduced to 15 principal components based on a-scores; and 2) a discriminant analysis step, where two discriminants are used to identify the linear combination of principal components from the first step that best distinguished prior groupings (populations) of individuals. The use of this multivariate approach is complementary to the STRUCTURE analysis, because of its ability to identify genetic structure in large databases without assumptions on the underlying genetic model. Thus, it is particularly suitable to identify variation between groups, while overlooking within-group variation. On the other hand, since DAPC does not specifically model for admixture, it is not suitable to identify individuals of mixed origin [40].

To measure the amount of genetic divergence among sampling localities, and the inferred genetic clusters and sampling sites, pairwise F_ST_ values and associated P values were calculated using ARLEQUIN v3.5 [41]. FST is another F-statistic measure (see above) and measures the proportion of the total genetic variance contained in a subpopulation. It ranges from 0 to 1, with high FST implying a considerable degree of differentiation among populations. Calculations to test for the statistical significance of the FST values were performed for 10,000 permutations. The same software was used to carry out a hierarchical analysis of molecular variance (AMOVA) to analyze the partitioning of the genetic variance (a) among and within the genetic clusters detected using previously described methods, (b) among and between three pre-defined groups within each genetic cluster: host (human, cattle, sheep, pig, dog, wild animals and tsetse flies), time of isolation, subspecies, and (c) among all samples based on date of collection. Samples were grouped at different time intervals (1 year, 5 years, 10 years) of collection to determine whether observed genetic variation could be attributed to temporal turnover. Each AMOVA analysis was run for 10,000 permutations with an allowable missing data level of 40%.

We used the LD bias correction method [42] implemented in LDNe [43] to estimate the effective population size (*Ne*) of each genetic cluster. We ran the analysis using a lowest allele frequency of 0.01.

## Results

### Taxon identification and genetic diversity

Of the 269 *T*. *brucei* isolates analyzed, 210 (78%) were *Tbr*, as determined by the presence of the *SRA* gene. While the majority of *SRA* positive samples were found among human isolates, 32% (21/69) of isolates from non-human vertebrate hosts tested positive for the *SRA* gene (S3 Table), indicating that *Tbr* strains are circulating in these animals with cattle forming the largest proportion (16 of 21; 76%).

The final dataset for analysis included samples from 19 districts in Uganda and Kenya (Fig. 1), averaging 13 samples per district. The average amplification rate was 70.0% across the 17 microsatellite loci (S.E. 12.13%); the 2010/2011 field samples collected on FTA cards had variable template concentration, leading to non-amplification due to low template concentration [28]. Only two loci (Tryp66 and Tryp5_2) out of 136 pairwise comparisons showed significant values (p>0.5; S4 Table), thus suggesting that they are in linkage disequilibrium. However, as expected, due to clonal reproduction in *T*. *brucei*, all loci deviated from HWE in at least one district (S5 Table). Levels of genetic diversity were within the norm observed for diploid outbreeding organisms (Table 1). Allelic richness (A_R_) ranged between 2.24 and 7.35 (districts for which a single sample was collected were excluded; Table 1). Similarly, heterozygosity levels were within the norm (H_E_ ranged from 0.34 to 0.70, H_O_ from 0.27 to 0.57). F_IS_ values were not high, ranging from -0.16 to 0.43 (Table 1), suggesting that inbreeding is not a major issue in this dataset. All genotypic data are submitted to *Dryad* (http://datadryad.org); DOI: doi:10.5061/dryad.m7q4c) [55].

**Table 1:** Sampling locality details.

Sampling site	Symbol	Country	N	A_R_	H_E_	H_O_	F_IS_
Apach	AP	Uganda	1	1.2	N/A	N/A	N/A
Bukedae	BKD	Uganda	1	1.5	N/A	N/A	N/A
Bugiri	BG	Uganda	7	3.2	0.47	0.41	0.13
Busia	BU	Uganda/Kenya	32	3.4	0.35	0.28	0.20
Busoga	BS	Uganda	23	5.3	0.51	0.43	0.18
Dokolo	DK	Uganda	11	2.4	0.24	0.17	0.42
Kaberamaido	KA	Uganda	59	3.7	0.25	0.19	0.32
Kampala	KP	Uganda	1	1.7	N/A	N/A	N/A
Kayunga	KY	Uganda	2	2.4	0.75	0.75	-0.04
Kole	KO	Uganda	25	3.9	0.29	0.18	0.39
Lira	LR	Uganda	10	2.8	0.33	0.24	0.26
Mukono	MK	Uganda	3	2.2	0.60	0.64	-0.16
Pallisa	PL	Uganda	15	2.4	0.30	0.37	-0.17
Soroti	SR	Uganda	25	4.2	0.39	0.24	0.43
Tororo	TR	Uganda	31	4.7	0.47	0.45	0.11
Teso	TS	Kenya	1	1.4	N/A	N/A	N/A
Central Nyanza	CN	Kenya	9	2.1	0.41	0.55	-0.25
South Nyanza	SN	Kenya	10	2.3	0.36	0.28	0.24
Sidende	SD	Kenya	1	N/A	N/A	N/A	N/A

Sample sizes and genetic diversity statistics for seventeen microsatellite loci across *Trypanosoma brucei* isolates from 19 districts (Fig. 1). N = number of samples analyzed, A_R_ = allele richness, H_E_ = expected heterozygosity, H_O_ = observed heterozygosity and F_IS_ = Fisher’s inbreeding coefficient. N/A = data not available because only a single sample was collected.

### Population structure, differentiation among groups, and Ne estimates

**Fig 2:**
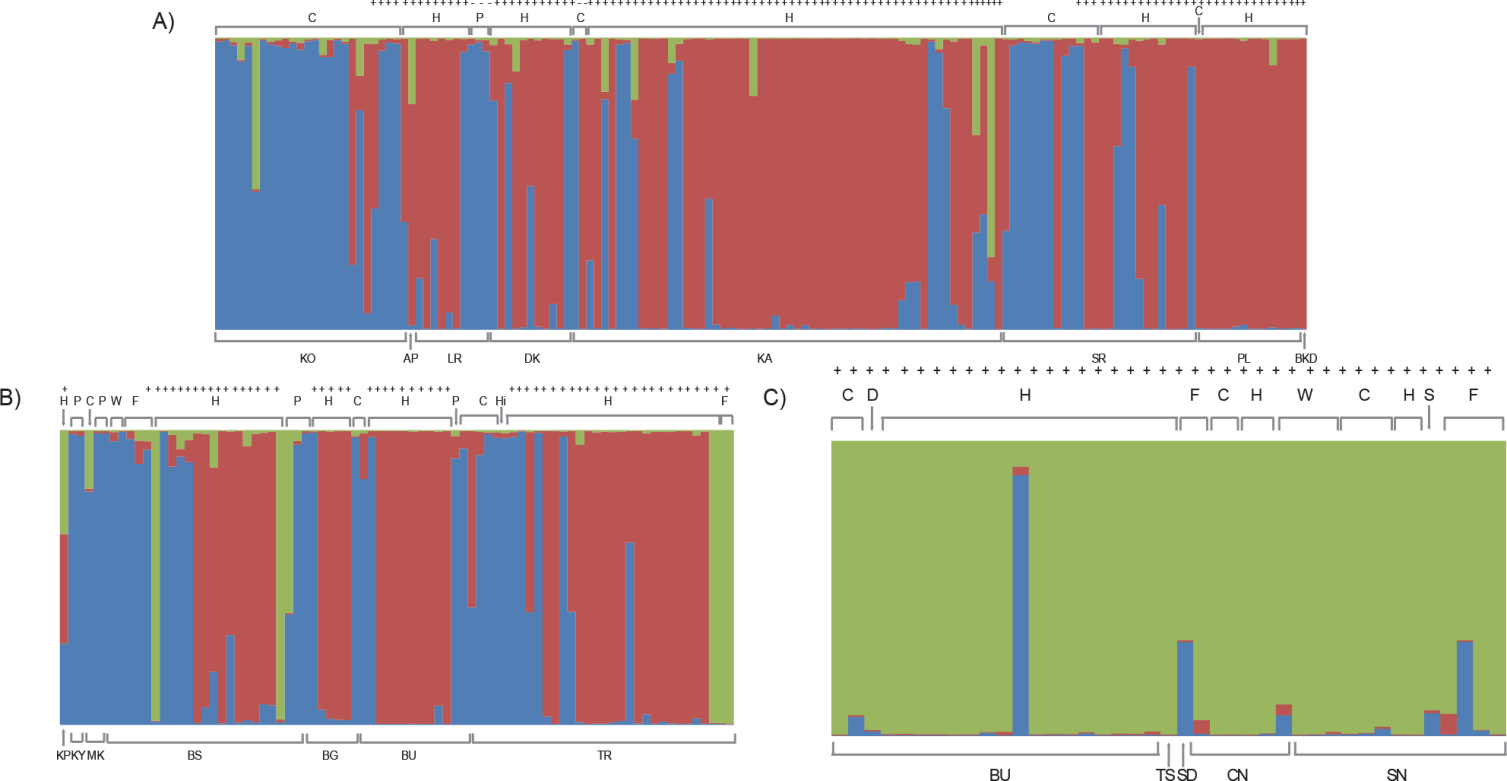
Population structure based on Bayesian clustering (ΔK = 3) for 269 samples of *Trypanosoma brucei brucei (Tbb)* and *Trypanosoma brucei rhodesiense (Tbr)* isolates from Uganda and Kenya, genotyped at 17 microsatellite loci. Samples are separated into three geographic regions as in Fig. 1. A) Central Uganda; B) Southern Uganda; C) Kenya. The district of origin of each sample is reported at the bottom of each panel (A-C), using the same abbreviations as in Table 1, a bracket line groups samples from the same district. Within each panel (A-C), samples are organized by districts. The districts are shown below each A-C plot in a west-east direction—with abbreviations corresponding with Table 1. Host is shown immediately above each plot (H = human, C = cattle, D = dog, P = pig, S = sheep, F = tsetse fly, W = Wildlife). Above the host information, + denotes samples with the *SRA* gene present. Each bar represents an isolate, the colors within the bar reflect the percent assignment (shown on the Y axis) of that individual to one of three genetic clusters (blue, green and red represent clusters 1–3, respectively). The proportion of each color in each individual represents the probability with which an individual is assigned to each of the three color-coded clusters. Individual assignment values (Q) to the three clusters are listed in Table 1.

**Fig 3:**
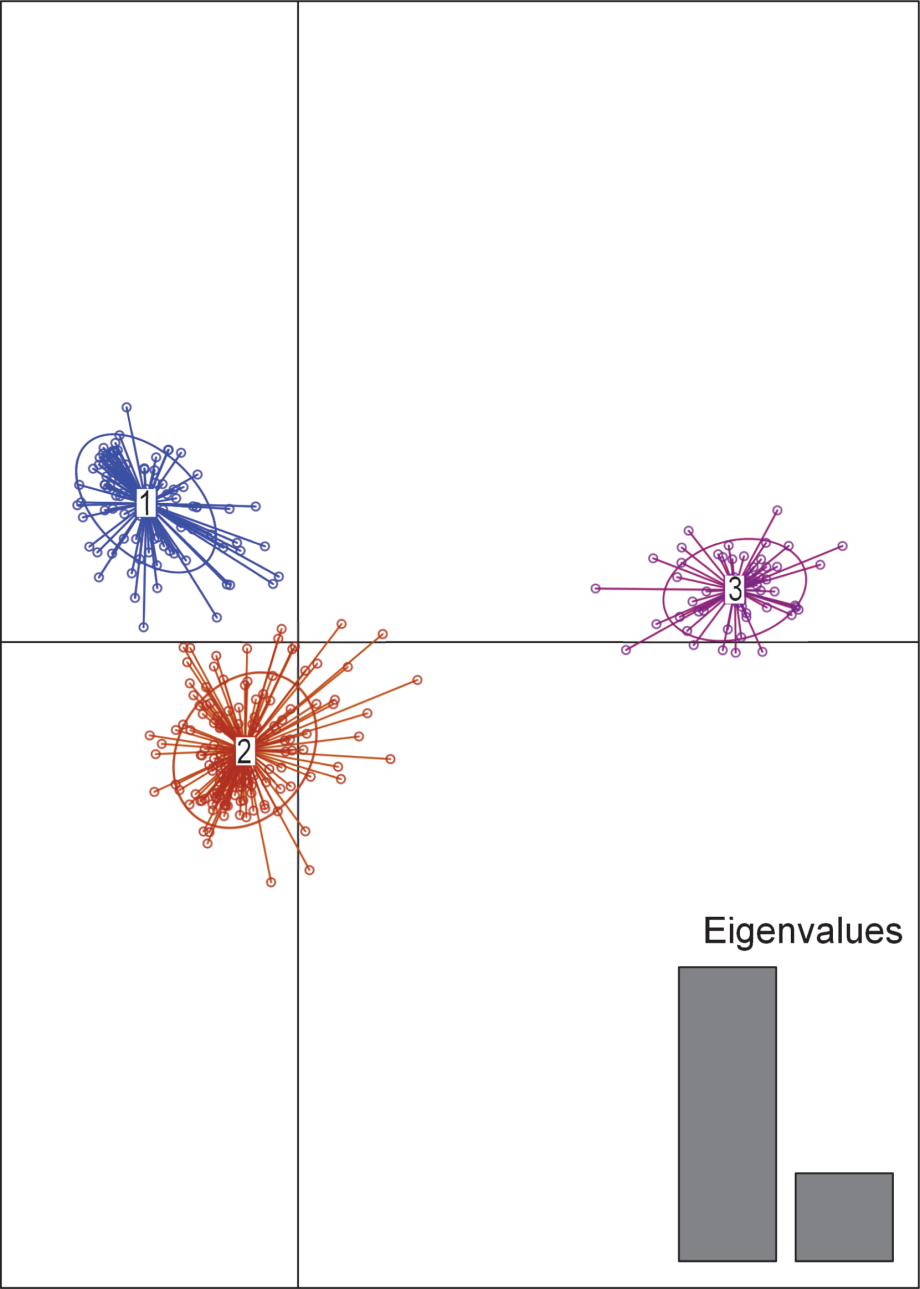
Discriminant analysis of principal components (DAPC). Two linear discriminants (LD1 and LD2) were used, following selection of principal components using a-score optimization, to plot *T*. *brucei* individual isolates. Dots represent individual genotypes connected by a line to the center of an ellipse with different colors representing the three clusters; blue (cluster 1), red (cluster 2), and purple (cluster 3).

Fig. 2, Table 1, and S1 Fig. show the results of the Bayesian clustering analyses as implemented in Structure; the 269 isolates are grouped in 3 genetic clusters (S1 Fig.). Clusters 1 and 3 as designated in Fig. 2 and S1 Table include isolates from mostly central and southeastern Uganda, while cluster 2 is mostly made up of isolates from Kenya. Besides geographic origin, Fig. 2 also shows the assignment of each isolate to one of the three clusters in relation to its host and taxonomic designation (*Tbr* vs *Tbb*, as assessed by the presence of the *SRA* gene). *Tbb* and *Tbr* samples are found together in clusters 1 and 3, indicating that *Tbr* strains are not genetically differentiated from the co-occurring *Tbb* strains; most isolates in cluster 2 were *SRA* positive. The results of the same analyses with samples grouped by collection date rather than geographic location is presented in S2 Fig. This Structure plot suggests that most of the early samples tend to belong to only two clusters (one and three), while samples from the early 1990’s mostly belong to the red and green cluster, although samples from the purple cluster still occur at these later dates. Interestingly the early samples were collected mostly from the Busia district in Uganda and Kenya. Temporal isolates from this region group in different clusters (Table 1), suggesting strain turnover in that region, although this analysis only shows a qualitative pattern (see results of the AMOVA analyses below). We also ran the same analyses omitting all the Kenyan samples to explore if without them we could detect additional subdivisions within the Uganda samples, but recovered only the same two clusters as in the analyses including all the samples (S3 Fig.). Note that the Structure results in Figs. 2 and 3 are not directly comparable, as the dataset and number of optimal clusters differ between the two analyses.

Within sampling sites, individuals with varying degrees of assignment to each of the three genetic clusters co-occur (S1 Table). This implies that, although the clusters are genetically distinct, genetic admixture is occurring. This is also evidence of recent long-range dispersal. An example of this phenomenon is the presence in a given sampling locality of (1) individuals with 100% assignment to a different genetic cluster than other samples from the same locality, and (2) genetically admixed individuals, likely the result of mating between local and immigrant strains. Importantly, localities in the southeastern (Busoga, BS, Busia, BU, Tororo, TR, Fig. 1), and central (Soroti, SR, Kaberamaido, KA, and Dokolo, DK, Fig. 1) Ugandan foci share strains from both cluster 1 and 3 (only one strain from cluster 2), implying that the strains from the old and new foci are not genetically distinct.

The southwestern Kenyan samples mostly belong to cluster 2 (Figs. 2 and 3), although a few individuals with genetic assignment to cluster 1 (blue bars in Fig. 2) can also be found in this region. Similarly, a few individuals from cluster 2 (both pure and admixed) can be found in central and southeastern Uganda, suggesting ongoing gene flow in both directions, even though most of the Kenyan and southern Uganda isolates belong to two different genetic clusters.

Fig. 3 shows the results of DAPC clustering of the same isolates, and confirms the identification of three distinct genetic clusters identified by the Bayesian based Structure analyses with the large majority of the individuals belonging to the same 3 clusters identified by structure. Table 1 reports the assignment of each isolate to the 3 clusters by both methods.

F_ST_ values between sampling sites ranged from 0 to 0.67 (S5 Table), and F_ST_ values between the three structure and DAPC inferred clusters ranged from 0.24 to 0.46 (S6 Table). The occurrence of statistically significant F_ST_ values among the three structure/DAPC inferred clusters confirms their genetic distinctiveness. The finding of relatively low and not statistically significant F_ST_ values among some of the isolates from different sampling sites and genetic clusters confirms the occurrence of genetic admixture also suggested by the Structure analysis (Fig. 2).

AMOVA results show the level of genetic diversity explained by the structure inferred genetic clusters and how much of the genetic variation is explained by collection date, species host, subspecies designation both among all the samples from the 19 sampling sites, regardless of their cluster assignation and within each of the three genetic clusters (Table 2). Most of the genetic variation was apportioned within (71.8%) rather than among the three Structure-defined clusters. Interestingly and contrary to the qualitative pattern shown in S2 Fig., very little of the observed genetic variation among the 19 sampling sites (8.49%) was explained by collection date (samples grouped in 10 year intervals), indicating that genetic variation in *T*. *brucei* is not explained by temporal turnover. This result was confirmed by carrying out the same analysis but grouping the samples at 1 and 5 year intervals (results not shown). Within the clusters, subspecies designation, date of collection, and host explained relatively little of the observed variation.

**Table 2:** AMOVA results.

	Within Groups	Among Groups	P value
**Clusters**	79.21%	20.79%	<.01*
**Sampling Dates**	91.51%	8.49%	<0.01*
**Cluster 1**			
Host	83.87%	16.13%	<0.01*
Date	87.39%	12.61%	<0.01*
Subspecies	87.64%	12.36%	<0.01*
**Cluster 2**			
Host	95.15%	4.85%	0.01*
Date	92.57%	7.43%	0.03*
Subspecies	N/A	N/A	N/A
**Cluster 3**			
Host	97.37%	2.63%	0.07
Date	99.23%	0.77%	0.07
Subspecies	83.75%	16.25%	<0.01*

Results of AMOVA analyses on seventeen microsatellite loci of *T*. *brucei* isolates partitioned into four groups: host (human, cattle, sheep, pig, dog, wild animals and tsetse flies), year of isolation (decade), subspecies, and structure/DAPC inferred genetic clusters. Asterisks denote comparisons with significant p values (<0.05).

Effective population size estimates (N_e_) calculated using LNDe [43] for the 3 clusters structure/DAPC inferred clusters are reported in Table 3 together with their confidence intervals. N_e_ were smaller in clusters 1 and 2 (13.1 and 8.1, respectively) than in cluster 3 (44.3; Table 3). As the confidence intervals around these estimates were relatively narrow, all clusters differed significantly (p<0.05) in effective population size. The small N_e_ suggests that clusters 1 and 2 represent clonal/rapid expansions, while the larger N_e_ observed for cluster 3 implies that isolates within this cluster have undergone more sexual reproduction and belong to an older established population than the isolates belonging to the other two clusters.

**Table 3:** Effective population size estimates.

Cluster	Ne	Lower and Upper C.I
1	9.7	(8.1–11.6)
2	2.3	(1.7–3.0)
3	86.4	(28.1–170.4)

Estimates of effective population size (Ne) calculated using LNDe (Waples and Do, 2008) among the three structure/DAPC genetic clusters. C.I = Confidence interval.

## Discussion

The aim of this study was to examine the pattern of genetic differentiation of *Tbb* and *Tbr* isolates in Uganda and western Kenya, to understand population structure and the modalities of parasite spread to help support sustainable control strategies for AAT and HAT in this region. Continent wide studies have already shown that *Tbr* and *Tbb* strains should not be treated as reproductively isolated taxa, as some *Tbb* strains are more closely related to *Tbr* strains than their conspecifics and *vice versa* [7]. The use of a larger number of highly variable microsatellite loci than in previous studies, coupled with a dense spatial and temporal sampling strategy, enabled us to identify three genetic partitions within the Uganda/Kenya *T*. *brucei* isolates that were not revealed by previous studies and the existence of ongoing gene flow between them (Figs. 2 and 3).

Two of the three clusters contain a mixture of Ugandan *Tbr* isolates from the old foci in the southeast and from the new foci in central districts, while the third cluster groups *Tbr* isolates from western Kenya. Thus, despite their geographic proximity and the widespread view that the Kenyan focus was an extension of that in southeast Uganda [44], the Ugandan and Kenyan *Tbr* populations seem to be genetically distinct, although there is evidence of genetic admixture likely via both long and short-range dispersal. From the earliest isoenzyme studies onwards, it has been clear that *Tbr* differs between geographically distant foci [2–4, 23–5], but more overlap might have been expected between these neighboring foci in Uganda and Kenya, which were in close contact via Lake Victoria [44]. One factor distinguishing HAT from the two areas is transmission by different tsetse species. HAT in the lakeshore region of southeast Uganda was originally transmitted by the *morsitans* group fly *G*. *pallidipes* [20, 44], and this fly was also the vector of HAT in South Nyanza, Kenya [3] and in Busia [44]; however, in the Alego outbreak of *Tbr* in Central Nyanza, Kenya, transmission was by the *palpalis* group fly *G*. *f*. *fuscipes* [45]. In Uganda, transmission of *Tbr* also switched to *G*. *f*. *fuscipes* as *Tbr* extended northwards into areas infested with this species from the mid 1970’s onwards [20] and *G*. *f*. *fuscipes* is regarded as the main HAT vector in Uganda [19, 46]. Therefore, the factor that led to genetic isolation of cluster 2 could be adaptation to transmission by a different tsetse vector, *G*. *pallidipes*. Our results clearly rule out the hypothesis that *Tbr* spread from its traditional focus in southeastern Uganda to western Kenya in the 1950’s along with *G*. *pallidipes* [44], and furthermore, *G*. *pallidipes* populations in Uganda and Kenya are genetically distinct [47].

Separate transmission cycles may also explain the partitioning of Ugandan *Tbr* isolates into two genetic clusters, despite the fact that they are now sympatric. *Glossina f*. *fuscipes* and *G*. *pallidipes* occupy different biomes, have different host-feeding preferences, and susceptibility to trypanosome infection. Therefore, *a priori*, divergence would be expected among the trypanosome populations adapted to transmission cycles involving either of these vectors, and a switch from transmission by *G*. *pallidipes* to *G*. *f*. *fuscipes*, as occurred in southeastern Uganda, would be expected to select for certain genotypes, while allowing the two divergent trypanosome populations to mix. It may also be significant that the *G*. *f*. *fuscipes* populations to the north and south of Lake Kyoga are genetically distinct [16, 17], implying that transmission cycles in the old and new foci were separate until trypanosomes were transferred via movement of infected humans and livestock.

In earlier studies, two *Tbr* genotypes circulating in the old foci were defined by isoenzyme profiles (zymodemes) and correlated with clinical presentation; the Zambezi zymodeme was associated with more chronic progression of HAT than the Busoga zymodeme [48]. Although Goodhead *et al* [24] found no simple correlation between zymodeme designation and clade based on 11 microsatellite loci, some population sub-structuring was evident in their analysis, and perhaps inaccuracy in zymodeme classification, which is based on relatively few informative isoenzyme loci, has obscured the relationship. Goodhead *et al* [24] also compared the genome sequences of one representative Busoga and Zambezi isolate and found that, although the genomes were >99.8% identical, they showed extensive chromosome-wide SNP variation. Comparison with *Tbb* or *Tbg* genomes revealed that some chromosomes were mosaics of shared alleles, suggesting that the Ugandan *Tbr* strains might have originated through a hybridization event between *T*. *brucei* of East and West African origin. Historically it is known that *Tbg* was present in the lakeshore region of southeast Uganda in the early 20^th^ century, so it is indeed possible that introgression has occurred.

Previous studies showed that there is sub-structuring in trypanosome populations in relation to host and geography suggesting that both geography and host play a role in shaping the patterns of genetic differentiation among *Tbb* and *Tbr* isolates [23–4, 49]. Our study does not support this. Although the estimates of genetic differentiation among sampling sites are statistically significant for a number of pairwise comparisons (S5 Table), the biological significance of this result is questionable, given the AMOVA results from Table 2 which show that within each of the three genetic clusters taxonomy, date of collection, and host explain less than 16% of the overall observed genetic variation. However, it should also be noted that the results from the AMOVA analyses are somewhat weakened by the fact that the representation of time and space points or hosts is not uniform. To better address this aspect, a denser sampling scheme would have been appropriate. Unfortunately, while the spatial and host coverage could be improved by additional collections, which we plan to carry out, the temporal aspect of the study cannot be properly addressed, as additional collections are not available.

The finding of individuals of two genetic clusters in both the old and new Ugandan foci challenges previous studies [21, 24–5, 47, 49, 50], which suggested that *Tbr* isolates from the Ugandan old and new foci were genetically distinct. Our study, based on a much larger data set both in terms of loci and number of samples, and including both *Tbb* and *Tbr* co-occurring strains, suggests that the expansion of the disease to the new foci in central and western Uganda occurred from *Tbr* isolates spreading from the old to the new foci. This result is similar to what has been described to explain the spread of HAT in Tanzania, showing genetic homogeneity of *Tbr* isolates in the region [51]. In addition, estimates of Ne (Table 3) show that clusters 1 and 2 have much lower effective population sizes than cluster 3, indicating that clusters 1 and 2 experienced recent clonal expansion, whereas cluster 3 had a higher rate of sexual reproduction. This may also explain the discord between our results and those of others.

Our results concur with previous studies that identified *Tbr* epidemics involving multiple lineages [3, 20], since *Tbr* strains with different genetic background co-occur in both the new and the old foci (Figs 2 and 3). We found no evidence for temporal structure in Ugandan *T*. *brucei*, whereas Duffy *et al* [25] found evidence of genetic shifts in allelic frequencies between samples collected in 1970 and 1990, as well as very low genetic similarity between samples from the old and new Ugandan foci. Here, temporal variation does not explain the partitioning of the observed genetic variation as shown by the AMOVA (Table 2) analyses and by the occurrence in the same genetic clusters of samples collected at different time points from the same or different sampling sites (Fig. 2 and 3). Instead we found more evidence of geographic genetic structuring (S5 Table). In this sense our study parallels better the Duffy *et al* [25] result for the Malawi strains rather than Uganda ones, underscoring the importance of using highly variable markers for studies such as this, where genetic differentiation levels are expected to be small, given the narrow spatial and temporal scale of the study. The other important difference between these studies that may play a role in explaining the different results is that the Duffy *et al* [25] study was entirely focused on *Tbr* strains from human patients, while our study looked at the genetic differentiation of co-occurring *Tbb* and *Tbr* isolates and included 32% of *Tbr* strains from non-human isolates. Looking at the whole spectrum of circulating genotypes provides additional insights on the evolutionary origin of the strains and their level of genetic admixture, as this and other studies have clearly shown that *Tbr* strains originate from *Tbb* strains, when they acquire the *SRA* gene [7].

We agree with Duffy *et al* [25] that the clonal nature of *T*. *brucei* may play a very important role in shaping its population dynamics. Our data show clear evidence of linkage disequilibrium at most loci with striking differences in effective population size estimates between clusters 1 and 2 *vs*. cluster 3 (Ne; Table 3), which could be an example of the potential for rapid population contractions and expansion of different genotypes due to clonal reproduction. A phenomenon that can happen stochastically and could be responsible for the different N_e_ estimate for clusters 1 and 2 *vs* cluster 3.

Our results also confirm that cattle are an important reservoir for *Tbr* and thus are likely to fuel the epidemiology of sleeping sickness in Uganda as 28.1% of the *T*. *brucei* isolates found in cattle (16/57) this study were *SRA* positive (S1 Table). Importantly, in the Structure analyses they clustered together with the human isolates from the same geographic regions (Fig. 2), suggesting ongoing genetic exchange between *T*. *brucei* isolates from cattle and humans in the same area. This result provides the first empirical confirmation that cattle are an important intermediary in HAT transmission in the region [23], as suggested in connection with earlier *Tbr* outbreaks [3, 44] and more recently for the current epidemics in Soroti and Kaberamaido [11, 52]. As the distance separating the *Tbr* and *Tbg* foci in North western Uganda is less than 100 km [11, 14], understanding the role and impact of cattle in fueling movement of *Tbr* strains is of paramount importance, as these results suggest that continued cattle movement from southern districts can expedite the fusion of the two disease belts with unknown public health consequences. Similarly, increased livestock trade across southeastern Uganda and Western Kenya also poses a risk transferring *Tbr* from the old Uganda HAT foci in that region to Western Kenya, which has been reporting low HAT prevalence in the last decade [53–54].

In conclusion, this study shows that there is genetic structuring within *T*. *brucei* populations from Uganda and Kenya, separating the isolates into three groups. We found clear evidence of ongoing genetic admixture and long-range dispersal among *Tbb* and *Tbr* strains. The use of a dense sampling scheme and highly variable loci enabled us to detect genetic exchange between the old and new Uganda disease foci, possibly mediated by cattle movements across the region as both *Tbb* and *Tbr* strains were found circulating in cattle. These results have important implications for disease control, as they provide empirical evidence for the occurrence of genetic exchange between co-occurring human infective and non-infective strains, and the role of cattle in spreading the human disease. The study also emphasizes the importance of studying both *Tbb* and *Tbr* strains when attempting to understand the population dynamics of *Tbr*.

## Supporting Information

S1 Table Details of the 269 *Tbb* and *Tbr* samples used in the study. The first three columns list the sample name, and its geographic origin (Country and District). The fourth column shows the code used in this study to identify a district. The following columns identify the named subspecies for each isolate (Taxon), the presence/absence of the SRA gene (SRA), the isolate host (Host), and the year of collection (Year). The next three columns report the Q values (the probability an individual to be assigned to each of the three clusters detected by the Structure analysis). The last column report the individual assignment based on the DAPC analysis.
(DOCX)

S2 Table Information on microsatellite loci and primers used in the analyses. The first two columns report the locus name. The next two columns show the DNA sequence of the forward and reverse primers, specifying in parenthesis the type of fluorescent dye used for each one. The next two columns list the repeat motif for each locus and the range of length of the alleles in base pairs (bp). The second to the last column reports the chromosomal location of each locus according to the reference in the last column.
(DOCX)

S3 Table Results of ITS and SRA screening of animal trypanosome isolates. The first three columns list the geographic origin (Origin), the district abbreviations (Code), and the host (Host). The forth column shows the number of strains for each host (N). The following three columns provide information on the *Trypanosoma* species infection in the samples other than *T. brucei* (*T. vivax = Tv; T, congolense*) and the occurrence of mixed *Tv* and *Tc* infections. The next two columns summarize the number of *Tbb* and *Tbr* samples, according to the SRA test. The final column reports the number of samples that did not produce PCR products, likely due to low DNA concentration and/or poor quality.
(DOCX)

S4 Table Linkage disequilibrium (LD) for all pairs of the seventeen microsatellites tested at 10,000 permutations in Arlequin (Excoffier et al., 2005). P-values and their Standard Errors (S.E>) are reported in the last column
(DOCX)

S5 Table Average pairwise FST (Weir and Cockerham 1984) values among 19 *Trypanosoma brucei* sampling sites obtained using Arlequin (Excoffier et al., 2005) and averaged across 17 loci. Asterisks denote statistically significant values (*P<0.05; **P<0.01).
(DOCX)

S6 Table Pairwise FST estimation among the three *Trypanosoma brucei* genetic structure/DAPC inferred clusters (1, 2 and 3) estimated using Arlequin (Excoffier et al., 2005) and averaged across 17 loci. Two asterisks indicate significance at P<0.01.
(DOCX)

S1 Fig Estimation of population clustering level from *Trypanosoma brucei* microsatellite genotypes following Evanno et al 2005 criteria. The highest peak at ΔK represents the most appropriate number of genetic clusters (K = 3).
(JPG)

S2 Fig Results of Bayesian clustering conducted in STRUCTURE arranged by date of collection. Dark bold vertical lines group the samples by decade of collections from 1970’s to 2010’s. Numbers on the vertical axis (0–1) refer to the individual assignment of each sample the three genetic clusters (red, purple, and green).
(JPG)

S3 Fig Results of Bayesian clustering conducted in STRUCTURE of only Ugandan samples. Dark bold vertical lines group the samples by district (symbol for each district is listed above the plot. The most likely number of cluster is 2 (K = 2). The vertical axis represents the individual assignment of each sample the two inferred clusters (red, blue).
(PDF)
