# Supplementary material for: Genetic Diversity and Population Structure of Trypanosoma brucei in Uganda: Implications for the Epidemiology of Sleeping Sickness and Nagana
Source: PLoS Negl Trop Dis. 2015 Feb 19;9(2):e0003353. doi: 10.1371/journal.pntd.0003353 (PMC4335064; doi:10.1371/journal.pntd.0003353)
Supplement: S1 Table — The first three columns list the sample name, and its geographic origin (Country and District). The fourth column shows the code used in this study to identify a district. The following columns identify the named subspecies for each isolate (Taxon), the presence/absence of the SRA gene (SRA), the isolate host (Host), and the year of collection (Year). The next three columns report the Q values (the probability an individual to be assigned to each of the three clusters detected by the Structure analysis). The last column report the individual assignment based on the DAPC analysis. (DOCX) [file pntd.0003353.s001.docx]

|  |  |  |  |  |  |  |  | **Structure Clusters** | | |  |
| --- | --- | --- | --- | --- | --- | --- | --- | --- | --- | --- | --- |
| **Sample ID** | **Country** | **District** | **Code** | **Taxon** | **SRA** | **Host** | **Year** | **1** | **2** | **3** | **DAPC cluster** |
| H120 | Uganda | Apac | AP | *Tbr* | + | Human | 2010 | 0.369 | 0.003 | 0.628 | 3 |
| H573 | Uganda | Bugiri | BG | *Tbb* | - | Pig | 2001 | 0.989 | 0.004 | 0.007 | 1 |
| H588 | Uganda | Bugiri | BG | *Tbb* | - | Pig | 2001 | 0.990 | 0.005 | 0.005 | 1 |
| H839 | Uganda | Bugiri | BG | *Tbr* | ₊ | Human | 1977 | 0.976 | 0.020 | 0.004 | 1 |
| H592 | Uganda | Bugiri | BG | *Tbr* | ₊ | Human | 1990 | 0.021 | 0.005 | 0.974 | 3 |
| H593 | Uganda | Bugiri | BG | *Tbr* | ₊ | Human | 1990 | 0.017 | 0.004 | 0.979 | 3 |
| H594 | Uganda | Bugiri | BG | *Tbr* | ₊ | Human | 1990 | 0.017 | 0.003 | 0.981 | 3 |
| H364 | Uganda | Bugiri | BG | *Tbr* | ₊ | Human | 2009 | 0.052 | 0.004 | 0.945 | 3 |
| H100 | Uganda | Bukedea | BKD | *Tbr* | ₊ | Human | 2011 | 0.003 | 0.003 | 0.994 | 3 |
| H849 | Uganda | Busoga | BS | *Tbb* | - | *G.pallidipes* | 1969 | 0.933 | 0.038 | 0.029 | 1 |
| H201 | Uganda | Busoga | BS | *Tbb* | - | *G.f.f* | 1971 | 0.970 | 0.004 | 0.027 | 1 |
| H291 | Uganda | Busoga | BS | *Tbb* | - | *G.pallidipes* | 1969 | 0.886 | 0.036 | 0.078 | 1 |
| H851 | Uganda | Busoga | BS | *Tbb* | - | Pig | 1991 | 0.950 | 0.037 | 0.013 | 1 |
| H832 | Uganda | Busoga | BS | *Tbb* | - | Wildlife | 1966 | 0.962 | 0.007 | 0.031 | 1 |
| H262 | Uganda | Busoga | BS | *Tbb* | - | Wildlife | 1970 | 0.994 | 0.003 | 0.003 | 1 |
| H243 | Uganda | Busoga | BS | *Tbr* | ₊ | Human | 1959 | 0.994 | 0.003 | 0.003 | 1 |
| H646 | Uganda | Busoga | BS | *Tbr* | ₊ | Human | 1960 | 0.891 | 0.034 | 0.075 | 1 |
| H303 | Uganda | Busoga | BS | *Tbr* | ₊ | Human | 1961 | 0.876 | 0.017 | 0.107 | 1 |
| H640 | Uganda | Busoga | BS | *Tbr* | ₊ | Human | 1961 | 0.910 | 0.064 | 0.026 | 1 |
| Try055 | Uganda | Busoga | BS | *Tbr* | ₊ | Human | 1972 | 0.374 | 0.621 | 0.005 | 2 |
| H837 | Uganda | Busoga | BS | *Tbr* | ₊ | Human | 1976 | 0.180 | 0.127 | 0.693 | 3 |
| Try009 | Uganda | Busoga | BS | *Tbr* | ₊ | Human | 1976 | 0.008 | 0.981 | 0.011 | 2 |
| H834 | Uganda | Busoga | BS | *Tbr* | ₊ | Human | 1979 | 0.006 | 0.011 | 0.983 | 3 |
| H841 | Uganda | Busoga | BS | *Tbr* | ₊ | Human | 1991 | 0.005 | 0.003 | 0.992 | 3 |
| H843 | Uganda | Busoga | BS | *Tbr* | ₊ | Human | 1991 | 0.305 | 0.005 | 0.691 | 3 |
| H844 | Uganda | Busoga | BS | *Tbr* | ₊ | Human | 1991 | 0.008 | 0.003 | 0.989 | 3 |
| H845 | Uganda | Busoga | BS | *Tbr* | ₊ | Human | 1991 | 0.016 | 0.054 | 0.929 | 3 |
| H846 | Uganda | Busoga | BS | *Tbr* | ₊ | Human | 1993 | 0.009 | 0.013 | 0.979 | 3 |
| H848 | Uganda | Busoga | BS | *Tbr* | ₊ | Human | 1993 | 0.070 | 0.010 | 0.920 | 3 |
| H836 | Uganda | Busoga | BS | *Tbr* | ₊ | Human |  | 0.060 | 0.013 | 0.927 | 3 |
| H853 | Uganda | Busoga | BS | *Tbr* | ₊ | Human |  | 0.066 | 0.006 | 0.928 | 3 |
| Try045 | Uganda | Busoga | BS | *Tbr* | ₊ | Tsetse fly | 1963 | 0.012 | 0.985 | 0.003 | 2 |
| H581 | Uganda | Busia | BU | *Tbb* | - | Cattle | 2009 | 0.834 | 0.011 | 0.156 | 1 |
| H879 | Uganda | Busia | BU | *Tbb* | - | Cattle | 2009 | 0.976 | 0.004 | 0.020 | 1 |
| H558 | Uganda | Busia | BU | *Tbb* | - | Pig | 2007 | 0.938 | 0.003 | 0.060 | 1 |
| Try019 | Kenya | Busia | BU | *Tbr* | ₊ | Cattle | 1987 | 0.002 | 0.995 | 0.003 | 2 |
| Try020 | Kenya | Busia | BU | *Tbr* | ₊ | Cattle | 1995 | 0.066 | 0.929 | 0.006 | 2 |
| Try013 | Kenya | Busia | BU | *Tbr* | ₊ | Dog | 2001 | 0.016 | 0.979 | 0.005 | 2 |
| Try005 | Kenya | Busia | BU | *Tbr* | ₊ | Human | 1989 | 0.003 | 0.993 | 0.004 | 2 |
| H598 | Uganda | Busia | BU | *Tbr* | ₊ | Human | 1990 | 0.003 | 0.003 | 0.994 | 3 |
| H599 | Uganda | Busia | BU | *Tbr* | ₊ | Human | 1990 | 0.003 | 0.003 | 0.994 | 3 |
| H600 | Uganda | Busia | BU | *Tbr* | ₊ | Human | 1990 | 0.003 | 0.003 | 0.994 | 3 |
| H602 | Uganda | Busia | BU | *Tbr* | ₊ | Human | 1990 | 0.066 | 0.006 | 0.928 | 3 |
| H619 | Uganda | Busia | BU | *Tbr* | ₊ | Human | 1990 | 0.003 | 0.003 | 0.994 | 3 |
| H865 | Uganda | Busia | BU | *Tbr* | ₊ | Human | 1990 | 0.905 | 0.020 | 0.075 | 3 |
| H869 | Uganda | Busia | BU | *Tbr* | ₊ | Human | 1990 | 0.003 | 0.003 | 0.994 | 3 |
| H577 | Uganda | Busia | BU | *Tbr* | ₊ | Human | 1991 | 0.003 | 0.003 | 0.994 | 3 |
| H610 | Uganda | Busia | BU | *Tbr* | ₊ | Human | 1991 | 0.004 | 0.006 | 0.990 | 3 |
| H611 | Uganda | Busia | BU | *Tbr* | ₊ | Human | 1991 | 0.003 | 0.003 | 0.994 | 3 |
| Try006 | Kenya | Busia | BU | *Tbr* | ₊ | Human | 1997 | 0.003 | 0.992 | 0.005 | 2 |
| Try054 | Kenya | Busia | BU | *Tbr* | ₊ | Human | 1997 | 0.005 | 0.984 | 0.011 | 2 |
| Try028 | Kenya | Busia | BU | *Tbr* | ₊ | Human | 1999 | 0.003 | 0.993 | 0.004 | 2 |
| Try007 | Kenya | Busia | BU | *Tbr* | ₊ | Human | 2000 | 0.003 | 0.994 | 0.003 | 2 |
| Try031 | Kenya | Busia | BU | *Tbr* | ₊ | Human | 2000 | 0.003 | 0.991 | 0.006 | 2 |
| Try032 | Kenya | Busia | BU | *Tbr* | ₊ | Human | 2000 | 0.003 | 0.992 | 0.005 | 2 |
| Try026 | Kenya | Busia | BU | *Tbr* | ₊ | Human | 2001 | 0.003 | 0.994 | 0.003 | 2 |
| Try029 | Kenya | Busia | BU | *Tbr* | ₊ | Human | 2001 | 0.009 | 0.987 | 0.004 | 2 |
| Try008 | Kenya | Busia | BU | *Tbr* | ₊ | Human | 2002 | 0.003 | 0.993 | 0.004 | 2 |
| Try021 | Kenya | Busia | BU | *Tbr* | ₊ | Human | 2002 | 0.002 | 0.995 | 0.003 | 2 |
| Try022 | Kenya | Busia | BU | *Tbr* | ₊ | Human | 2002 | 0.003 | 0.994 | 0.003 | 2 |
| Try023 | Kenya | Busia | BU | *Tbr* | ₊ | Human | 2002 | 0.011 | 0.986 | 0.003 | 2 |
| Try024 | Kenya | Busia | BU | *Tbr* | ₊ | Human | 2002 | 0.003 | 0.985 | 0.012 | 2 |
| Try025 | Kenya | Busia | BU | *Tbr* | ₊ | Human | 2002 | 0.884 | 0.088 | 0.028 | 1 |
| Try027 | Kenya | Busia | BU | *Tbr* | ₊ | Human | 2002 | 0.003 | 0.993 | 0.004 | 2 |
| Try030 | Kenya | Busia | BU | *Tbr* | ₊ | Human | 2002 | 0.003 | 0.994 | 0.003 | 2 |
| Try015 | Kenya | Central Nyanza | CN | *Tbr* | ₊ | Cattle | 1964 | 0.002 | 0.995 | 0.003 | 2 |
| Try016 | Kenya | Central Nyanza | CN | *Tbr* | ₊ | Cattle | 1967 | 0.003 | 0.994 | 0.003 | 2 |
| Try002 | Kenya | Central Nyanza | CN | *Tbr* | ₊ | Human | 1961 | 0.002 | 0.995 | 0.003 | 2 |
| Try004 | Kenya | Central Nyanza | CN | *Tbr* | ₊ | Human | 1977 | 0.006 | 0.991 | 0.003 | 2 |
| Try052 | Kenya | Central Nyanza | CN | *Tbr* | ₊ | Human | 1977 | 0.002 | 0.995 | 0.003 | 2 |
| Try012 | Kenya | Central Nyanza | CN | *Tbr* | ₊ | Wildlife | 1958 | 0.071 | 0.892 | 0.037 | 2 |
| Try0 51 | Kenya | Central Nyanza | CN | *Tbr* | ₊ | Cattle | 1967 | 0.025 | 0.968 | 0.008 | 2 |
| Try033 | Kenya | Central Nyanza | CN | *Tbr* | ₊ | Tsetse fly | 1961 | 0.007 | 0.946 | 0.047 | 2 |
| Try0 50 | Kenya | Central Nyanza | CN | *Tbr* | ₊ | Wildlife | 1958 | 0.006 | 0.985 | 0.009 | 2 |
| H458 | Uganda | Dokolo | DK | *Tbr* | ₊ | Human | 2009 | 0.003 | 0.115 | 0.882 | 3 |
| H460 | Uganda | Dokolo | DK | *Tbr* | ₊ | Human | 2009 | 0.009 | 0.009 | 0.982 | 3 |
| H467 | Uganda | Dokolo | DK | *Tbr* | ₊ | Human | 2009 | 0.492 | 0.004 | 0.505 | 3 |
| H472 | Uganda | Dokolo | DK | *Tbr* | ₊ | Human | 2009 | 0.010 | 0.010 | 0.980 | 3 |
| H624 | Uganda | Dokolo | DK | *Tbr* | ₊ | Human | 2009 | 0.005 | 0.005 | 0.990 | 3 |
| H111 | Uganda | Dokolo | DK | *Tbr* | ₊ | Human | 2010 | 0.784 | 0.026 | 0.190 | 1 |
| H114 | Uganda | Dokolo | DK | *Tbr* | ₊ | Human | 2010 | 0.003 | 0.003 | 0.994 | 3 |
| H117 | Uganda | Dokolo | DK | *Tbr* | ₊ | Human | 2010 | 0.844 | 0.023 | 0.133 | 1 |
| H493 | Uganda | Dokolo | DK | *Tbr* | ₊ | Human | 2010 | 0.003 | 0.003 | 0.994 | 3 |
| H511 | Uganda | Dokolo | DK | *Tbr* | ₊ | Human | 2010 | 0.088 | 0.005 | 0.907 | 3 |
| H109 | Uganda | Dokolo | DK | *Tbr* | ₊ | Human | 2011 | 0.953 | 0.008 | 0.039 | 1 |
| H170 | Uganda | Kaberamaido | KA | *Tbb* | - | Cattle | 2011 | 0.957 | 0.024 | 0.019 | 1 |
| RE12_058 | Uganda | Kaberamaido | KA | *Tbb* | - | Cattle |  | 0.989 | 0.007 | 0.004 | 1 |
| H498 | Uganda | Kaberamaido | KA | *Tbr* | ₊ | Human | 2006 | 0.003 | 0.003 | 0.994 | 3 |
| H356 | Uganda | Kaberamaido | KA | *Tbr* | ₊ | Human | 2009 | 0.006 | 0.004 | 0.990 | 3 |
| H359 | Uganda | Kaberamaido | KA | *Tbr* | ₊ | Human | 2009 | 0.005 | 0.005 | 0.991 | 3 |
| H360 | Uganda | Kaberamaido | KA | *Tbr* | ₊ | Human | 2009 | 0.085 | 0.013 | 0.901 | 3 |
| H361 | Uganda | Kaberamaido | KA | *Tbr* | ₊ | Human | 2009 | 0.017 | 0.003 | 0.980 | 3 |
| H362 | Uganda | Kaberamaido | KA | *Tbr* | ₊ | Human | 2009 | 0.003 | 0.003 | 0.994 | 3 |
| H459 | Uganda | Kaberamaido | KA | *Tbr* | ₊ | Human | 2009 | 0.003 | 0.002 | 0.995 | 3 |
| H462 | Uganda | Kaberamaido | KA | *Tbr* | ₊ | Human | 2009 | 0.004 | 0.200 | 0.796 | 3 |
| H463 | Uganda | Kaberamaido | KA | *Tbr* | ₊ | Human | 2009 | 0.002 | 0.003 | 0.995 | 3 |
| H464 | Uganda | Kaberamaido | KA | *Tbr* | ₊ | Human | 2009 | 0.004 | 0.004 | 0.992 | 3 |
| H465 | Uganda | Kaberamaido | KA | *Tbr* | ₊ | Human | 2009 | 0.049 | 0.004 | 0.947 | 3 |
| H468 | Uganda | Kaberamaido | KA | *Tbr* | ₊ | Human | 2009 | 0.004 | 0.003 | 0.993 | 3 |
| H470 | Uganda | Kaberamaido | KA | *Tbr* | ₊ | Human | 2009 | 0.016 | 0.004 | 0.980 | 3 |
| H474 | Uganda | Kaberamaido | KA | *Tbr* | ₊ | Human | 2009 | 0.333 | 0.334 | 0.333 | 3 |
| H104 | Uganda | Kaberamaido | KA | *Tbr* | ₊ | Human | 2010 | 0.004 | 0.003 | 0.993 | 3 |
| H103 | Uganda | Kaberamaido | KA | *Tbr* | ₊ | Human | 2010 | 0.237 | 0.023 | 0.739 | 3 |
| H110 | Uganda | Kaberamaido | KA | *Tbr* | ₊ | Human | 2010 | 0.654 | 0.213 | 0.133 | 1 |
| H112 | Uganda | Kaberamaido | KA | *Tbr* | ₊ | Human | 2010 | 0.949 | 0.039 | 0.012 | 1 |
| H113 | Uganda | Kaberamaido | KA | *Tbr* | ₊ | Human | 2010 | 0.005 | 0.002 | 0.993 | 3 |
| H115 | Uganda | Kaberamaido | KA | *Tbr* | ₊ | Human | 2010 | 0.004 | 0.004 | 0.992 | 3 |
| H116 | Uganda | Kaberamaido | KA | *Tbr* | ₊ | Human | 2010 | 0.004 | 0.003 | 0.993 | 3 |
| H118 | Uganda | Kaberamaido | KA | *Tbr* | ₊ | Human | 2010 | 0.003 | 0.003 | 0.995 | 3 |
| H119 | Uganda | Kaberamaido | KA | *Tbr* | ₊ | Human | 2010 | 0.759 | 0.006 | 0.236 | 1 |
| H121 | Uganda | Kaberamaido | KA | *Tbr* | ₊ | Human | 2010 | 0.876 | 0.087 | 0.037 | 1 |
| H122 | Uganda | Kaberamaido | KA | *Tbr* | ₊ | Human | 2010 | 0.921 | 0.021 | 0.058 | 1 |
| H123 | Uganda | Kaberamaido | KA | *Tbr* | ₊ | Human | 2010 | 0.007 | 0.005 | 0.988 | 3 |
| H125 | Uganda | Kaberamaido | KA | *Tbr* | ₊ | Human | 2010 | 0.003 | 0.002 | 0.995 | 3 |
| H128 | Uganda | Kaberamaido | KA | *Tbr* | ₊ | Human | 2010 | 0.449 | 0.007 | 0.544 | 3 |
| H129 | Uganda | Kaberamaido | KA | *Tbr* | ₊ | Human | 2010 | 0.018 | 0.003 | 0.979 | 3 |
| H130 | Uganda | Kaberamaido | KA | *Tbr* | ₊ | Human | 2010 | 0.004 | 0.003 | 0.993 | 3 |
| H131 | Uganda | Kaberamaido | KA | *Tbr* | ₊ | Human | 2010 | 0.007 | 0.003 | 0.990 | 3 |
| H461 | Uganda | Kaberamaido | KA | *Tbr* | ₊ | Human | 2010 | 0.002 | 0.003 | 0.995 | 3 |
| H476 | Uganda | Kaberamaido | KA | *Tbr* | ₊ | Human | 2010 | 0.101 | 0.012 | 0.887 | 3 |
| H478 | Uganda | Kaberamaido | KA | *Tbr* | ₊ | Human | 2010 | 0.003 | 0.002 | 0.996 | 3 |
| H479 | Uganda | Kaberamaido | KA | *Tbr* | ₊ | Human | 2010 | 0.015 | 0.003 | 0.982 | 3 |
| H481 | Uganda | Kaberamaido | KA | *Tbr* | ₊ | Human | 2010 | 0.395 | 0.027 | 0.579 | 3 |
| H483 | Uganda | Kaberamaido | KA | *Tbr* | ₊ | Human | 2010 | 0.162 | 0.022 | 0.816 | 3 |
| H485 | Uganda | Kaberamaido | KA | *Tbr* | ₊ | Human | 2010 | 0.165 | 0.752 | 0.084 | 2 |
| H487 | Uganda | Kaberamaido | KA | *Tbr* | ₊ | Human | 2010 | 0.165 | 0.025 | 0.810 | 3 |
| H489 | Uganda | Kaberamaido | KA | *Tbr* | ₊ | Human | 2010 | 0.003 | 0.003 | 0.994 | 3 |
| H490 | Uganda | Kaberamaido | KA | *Tbr* | ₊ | Human | 2010 | 0.004 | 0.003 | 0.993 | 3 |
| H491 | Uganda | Kaberamaido | KA | *Tbr* | ₊ | Human | 2010 | 0.002 | 0.002 | 0.996 | 3 |
| H492 | Uganda | Kaberamaido | KA | *Tbr* | ₊ | Human | 2010 | 0.003 | 0.003 | 0.994 | 3 |
| H495 | Uganda | Kaberamaido | KA | *Tbr* | ₊ | Human | 2010 | 0.002 | 0.002 | 0.996 | 3 |
| H505 | Uganda | Kaberamaido | KA | *Tbr* | ₊ | Human | 2010 | 0.004 | 0.002 | 0.994 | 3 |
| RE045 | Uganda | Kaberamaido | KA | *Tbr* | ₊ | Human | 2010 | 0.003 | 0.003 | 0.994 | 3 |
| RE12_045 | Uganda | Kaberamaido | KA | *Tbr* | ₊ | Human | 2010 | 0.986 | 0.006 | 0.008 | 1 |
| H101 | Uganda | Kaberamaido | KA | *Tbr* | ₊ | Human | 2011 | 0.003 | 0.003 | 0.994 | 3 |
| H105 | Uganda | Kaberamaido | KA | *Tbr* | ₊ | Human | 2011 | 0.789 | 0.185 | 0.026 | 1 |
| H106 | Uganda | Kaberamaido | KA | *Tbr* | ₊ | Human | 2011 | 0.003 | 0.003 | 0.994 | 3 |
| H107 | Uganda | Kaberamaido | KA | *Tbr* | ₊ | Human | 2011 | 0.976 | 0.007 | 0.017 | 1 |
| H108 | Uganda | Kaberamaido | KA | *Tbr* | ₊ | Human | 2011 | 0.981 | 0.008 | 0.011 | 1 |
| H124 | Uganda | Kaberamaido | KA | *Tbr* | ₊ | Human | 2011 | 0.003 | 0.003 | 0.994 | 3 |
| H351 | Uganda | Kaberamaido | KA | *Tbr* | ₊ | Human |  | 0.003 | 0.002 | 0.995 | 3 |
| H353 | Uganda | Kaberamaido | KA | *Tbr* | ₊ | Human |  | 0.003 | 0.003 | 0.994 | 3 |
| H354 | Uganda | Kaberamaido | KA | *Tbr* | ₊ | Human |  | 0.002 | 0.002 | 0.996 | 3 |
| H355 | Uganda | Kaberamaido | KA | *Tbr* | ₊ | Human |  | 0.003 | 0.003 | 0.994 | 3 |
| H014 | Uganda | Kole | KO | *Tbb* | - | Cattle | 2010 | 0.986 | 0.004 | 0.010 | 1 |
| H015 | Uganda | Kole | KO | *Tbb* | - | Cattle | 2010 | 0.990 | 0.004 | 0.006 | 1 |
| H017 | Uganda | Kole | KO | *Tbb* | - | Cattle | 2010 | 0.974 | 0.016 | 0.011 | 1 |
| H018 | Uganda | Kole | KO | *Tbb* | - | Cattle | 2010 | 0.918 | 0.076 | 0.006 | 1 |
| H025 | Uganda | Kole | KO | *Tbb* | - | Cattle | 2010 | 0.971 | 0.017 | 0.012 | 1 |
| H027 | Uganda | Kole | KO | *Tbb* | - | Cattle | 2010 | 0.473 | 0.520 | 0.008 | 1 |
| H029 | Uganda | Kole | KO | *Tbb* | - | Cattle | 2010 | 0.990 | 0.005 | 0.005 | 1 |
| H038 | Uganda | Kole | KO | *Tbb* | - | Cattle | 2010 | 0.977 | 0.014 | 0.009 | 1 |
| H045 | Uganda | Kole | KO | *Tbb* | - | Cattle | 2010 | 0.971 | 0.010 | 0.019 | 1 |
| H054 | Uganda | Kole | KO | *Tbb* | - | Cattle | 2010 | 0.965 | 0.004 | 0.031 | 1 |
| H055 | Uganda | Kole | KO | *Tbb* | - | Cattle | 2010 | 0.980 | 0.014 | 0.006 | 1 |
| H056 | Uganda | Kole | KO | *Tbb* | - | Cattle | 2010 | 0.959 | 0.018 | 0.023 | 1 |
| H065 | Uganda | Kole | KO | *Tbb* | - | Cattle | 2010 | 0.987 | 0.007 | 0.006 | 1 |
| H070 | Uganda | Kole | KO | *Tbb* | - | Cattle | 2010 | 0.992 | 0.003 | 0.005 | 1 |
| H073 | Uganda | Kole | KO | *Tbb* | - | Cattle | 2010 | 0.933 | 0.060 | 0.007 | 1 |
| H075 | Uganda | Kole | KO | *Tbb* | - | Cattle | 2010 | 0.933 | 0.009 | 0.058 | 1 |
| H085 | Uganda | Kole | KO | *Tbb* | - | Cattle | 2010 | 0.990 | 0.003 | 0.007 | 1 |
| H145 | Uganda | Kole | KO | *Tbb* | - | Cattle | 2010 | 0.979 | 0.021 | 0.007 | 1 |
| H152 | Uganda | Kole | KO | *Tbb* | - | Cattle | 2010 | 0.222 | 0.004 | 0.774 | 3 |
| H153 | Uganda | Kole | KO | *Tbb* | - | Cattle | 2010 | 0.752 | 0.131 | 0.117 | 1 |
| H019 | Uganda | Kole | KO | *Tbr* | ₊ | Cattle | 2010 | 0.056 | 0.021 | 0.923 | 3 |
| H031 | Uganda | Kole | KO | *Tbr* | ₊ | Cattle | 2010 | 0.415 | 0.007 | 0.564 | 3 |
| H034 | Uganda | Kole | KO | *Tbr* | ₊ | Cattle | 2010 | 0.955 | 0.004 | 0.038 | 1 |
| H095 | Uganda | Kole | KO | *Tbr* | ₊ | Cattle | 2010 | 0.983 | 0.014 | 0.013 | 1 |
| H151 | Uganda | Kole | KO | *Tbr* | ₊ | Cattle | 2010 | 0.979 | 0.005 | 0.016 | 1 |
| H621 | Uganda | Kampala | KP | *Tbr* | ₊ | Human | 2010 | 0.276 | 0.353 | 0.371 | 1 |
| H574 | Uganda | Kayunga | KY | *Tbb* | - | Pig | 2001 | 0.987 | 0.003 | 0.010 | 1 |
| H591 | Uganda | Kayunga | KY | *Tbb* | - | Pig | 2001 | 0.980 | 0.004 | 0.016 | 1 |
| H570 | Uganda | Lira | LR | *Tbb* | - | Pig | 2001 | 0.951 | 0.005 | 0.044 | 1 |
| H575 | Uganda | Lira | LR | *Tbb* | - | Pig | 2001 | 0.977 | 0.003 | 0.020 | 1 |
| H629 | Uganda | Lira | LR | *Tbb* | - | Pig | 2001 | 0.984 | 0.008 | 0.008 | 1 |
| H500 | Uganda | Lira | LR | *Tbr* | ₊ | Human | 2006 | 0.015 | 0.227 | 0.758 | 3 |
| H521 | Uganda | Lira | LR | *Tbr* | ₊ | Human | 2006 | 0.177 | 0.004 | 0.819 | 3 |
| H522 | Uganda | Lira | LR | *Tbr* | ₊ | Human | 2006 | 0.003 | 0.002 | 0.995 | 3 |
| H614 | Uganda | Lira | LR | *Tbr* | ₊ | Human | 2006 | 0.310 | 0.012 | 0.678 | 3 |
| H616 | Uganda | Lira | LR | *Tbr* | ₊ | Human | 2006 | 0.002 | 0.002 | 0.996 | 3 |
| H633 | Uganda | Lira | LR | *Tbr* | ₊ | Human | 2006 | 0.058 | 0.009 | 0.933 | 3 |
| H878 | Uganda | Lira | LR | *Tbr* | ₊ | Human | 2006 | 0.005 | 0.003 | 0.992 | 3 |
| H569 | Uganda | Mukono | MK | *Tbb* | - | Cattle | 2001 | 0.791 | 0.199 | 0.010 | 1 |
| H571 | Uganda | Mukono | MK | *Tbb* | - | Pig | 2001 | 0.989 | 0.004 | 0.007 | 1 |
| H576 | Uganda | Mukono | MK | *Tbb* | - | Pig | 2001 | 0.989 | 0.004 | 0.007 | 1 |
| H411 | Uganda | Pallisa | PL | *Tbb* | - | Cattle | 2009 | 0.900 | 0.004 | 0.096 | 1 |
| H335 | Uganda | Pallisa | PL | *Tbr* | ₊ | Human | 2008 | 0.003 | 0.003 | 0.994 | 3 |
| H339 | Uganda | Pallisa | PL | *Tbr* | ₊ | Human | 2008 | 0.003 | 0.003 | 0.994 | 3 |
| H340 | Uganda | Pallisa | PL | *Tbr* | ₊ | Human | 2008 | 0.003 | 0.003 | 0.994 | 3 |
| H341 | Uganda | Pallisa | PL | *Tbr* | ₊ | Human | 2008 | 0.003 | 0.002 | 0.995 | 3 |
| H613 | Uganda | Pallisa | PL | *Tbr* | ₊ | Human | 2008 | 0.004 | 0.005 | 0.991 | 3 |
| H872 | Uganda | Pallisa | PL | *Tbr* | ₊ | Human | 2008 | 0.008 | 0.095 | 0.897 | 3 |
| H873 | Uganda | Pallisa | PL | *Tbr* | ₊ | Human | 2008 | 0.004 | 0.005 | 0.991 | 3 |
| H345 | Uganda | Pallisa | PL | *Tbr* | ₊ | Human | 2009 | 0.011 | 0.003 | 0.986 | 3 |
| H346 | Uganda | Pallisa | PL | *Tbr* | ₊ | Human | 2009 | 0.017 | 0.010 | 0.972 | 3 |
| H348 | Uganda | Pallisa | PL | *Tbr* | ₊ | Human | 2009 | 0.003 | 0.003 | 0.994 | 3 |
| H875 | Uganda | Pallisa | PL | *Tbr* | ₊ | Human | 2009 | 0.004 | 0.005 | 0.991 | 3 |
| RE150 | Uganda | Pallisa | PL | *Tbr* | ₊ | Human | 2009 | 0.006 | 0.005 | 0.989 | 3 |
| H595 | Uganda | Pallisa | PL | *Tbr* | ₊ | Human | 2010 | 0.003 | 0.003 | 0.994 | 3 |
| H876 | Uganda | Pallisa | PL | *Tbr* | ₊ | Human | 2010 | 0.006 | 0.005 | 0.989 | 3 |
| Try010 | Uganda | Sidende | SD | *Tbr* | ₊ | Tsetse fly | 1970 | 0.319 | 0.675 | 0.006 | 2 |
| Try017 | Kenya | South Nyanza | SN | *Tbr* | ₊ | Cattle | 1970 | 0.005 | 0.992 | 0.003 | 2 |
| Try018 | Kenya | South Nyanza | SN | *Tbr* | ₊ | Cattle | 1980 | 0.008 | 0.989 | 0.003 | 2 |
| Try003 | Kenya | South Nyanza | SN | *Tbr* | ₊ | Human | 1969 | 0.002 | 0.994 | 0.004 | 2 |
| Try014 | Kenya | South Nyanza | SN | *Tbr* | ₊ | Sheep | 1970 | 0.077 | 0.912 | 0.011 | 2 |
| Try035 | Kenya | South Nyanza | SN | *Tbr* | ₊ | Tsetse fly | 1969 | 0.320 | 0.676 | 0.005 | 2 |
| Try037 | Kenya | South Nyanza | SN | *Tbr* | ₊ | Tsetse fly | 1969 | 0.002 | 0.994 | 0.004 | 2 |
| Try034 | Kenya | South Nyanza | SN | *Tbr* | ₊ | Tsetse fly | 1970 | 0.006 | 0.924 | 0.070 | 2 |
| Try036 | Kenya | South Nyanza | SN | *Tbr* | ₊ | Tsetse fly | 1981 | 0.019 | 0.978 | 0.003 | 2 |
| Try011 | Kenya | South Nyanza | SN | *Tbr* | ₊ | Wildlife | 1970 | 0.002 | 0.995 | 0.003 | 2 |
| Try048 | Kenya | South Nyanza | SN | *Tbr* | ₊ | Wildlife | 1970 | 0.004 | 0.993 | 0.003 | 2 |
| H541 | Uganda | Soroti | SR | *Tbb* | - | Cattle | 2003 | 0.006 | 0.006 | 0.988 | 3 |
| H547 | Uganda | Soroti | SR | *Tbb* | - | Cattle | 2003 | 0.938 | 0.003 | 0.059 | 1 |
| H864 | Uganda | Soroti | SR | *Tbb* | - | Cattle | 2003 | 0.971 | 0.003 | 0.026 | 1 |
| H446 | Uganda | Soroti | SR | *Tbb* | - | Cattle | 2009 | 0.338 | 0.005 | 0.657 | 3 |
| H527 | Uganda | Soroti | SR | *Tbb* | - | Cattle | Dates? | 0.973 | 0.007 | 0.020 | 1 |
| H528 | Uganda | Soroti | SR | *Tbb* | - | Cattle |  | 0.983 | 0.004 | 0.013 | 1 |
| H529 | Uganda | Soroti | SR | *Tbb* | - | Cattle |  | 0.982 | 0.011 | 0.007 | 1 |
| H531 | Uganda | Soroti | SR | *Tbb* | - | Cattle |  | 0.979 | 0.015 | 0.006 | 1 |
| H533 | Uganda | Soroti | SR | *Tbb* | - | Cattle |  | 0.990 | 0.006 | 0.004 | 1 |
| H540 | Uganda | Soroti | SR | *Tbb* | - | Cattle |  | 0.989 | 0.007 | 0.004 | 1 |
| H543 | Uganda | Soroti | SR | *Tbr* | ₊ | Cattle | 2003 | 0.972 | 0.019 | 0.010 | 1 |
| H625 | Uganda | Soroti | SR | *Tbr* | ₊ | Cattle | 2003 | 0.005 | 0.018 | 0.978 | 3 |
| H583 | Uganda | Soroti | SR | *Tbr* | ₊ | Cattle | 2008 | 0.002 | 0.002 | 0.996 | 3 |
| RE053 | Uganda | Soroti | SR | *Tbr* | ₊ | Human | 1999 | 0.002 | 0.002 | 0.996 | 3 |
| H585 | Uganda | Soroti | SR | *Tbr* | ₊ | Human | 2000 | 0.002 | 0.002 | 0.996 | 3 |
| H589 | Uganda | Soroti | SR | *Tbr* | ₊ | Human | 2001 | 0.002 | 0.002 | 0.996 | 3 |
| H590 | Uganda | Soroti | SR | *Tbr* | ₊ | Human | 2001 | 0.002 | 0.002 | 0.996 | 3 |
| H880 | Uganda | Soroti | SR | *Tbr* | ₊ | Human | 2003 | 0.004 | 0.002 | 0.994 | 3 |
| H586 | Uganda | Soroti | SR | *Tbr* | ₊ | Human | 2005 | 0.427 | 0.023 | 0.550 | 1 |
| H365 | Uganda | Soroti | SR | *Tbr* | ₊ | Human | 2009 | 0.003 | 0.002 | 0.995 | 3 |
| H367 | Uganda | Soroti | SR | *Tbr* | ₊ | Human | 2009 | 0.628 | 0.022 | 0.350 | 1 |
| H369 | Uganda | Soroti | SR | *Tbr* | ₊ | Human | 2009 | 0.962 | 0.025 | 0.013 | 1 |
| H515 | Uganda | Soroti | SR | *Tbr* | ₊ | Human | 2009 | 0.900 | 0.007 | 0.093 | 1 |
| H517 | Uganda | Soroti | SR | *Tbr* | ₊ | Human | 2009 | 0.174 | 0.011 | 0.815 | 3 |
| H519 | Uganda | Soroti | SR | *Tbr* | ₊ | Human | 2009 | 0.004 | 0.005 | 0.991 | 3 |
| H854 | Uganda | Tororo | TR | *Tbb* | - | Cattle | 1988 | 0.972 | 0.011 | 0.017 | 1 |
| H857 | Uganda | Tororo | TR | *Tbb* | - | Cattle | 1988 | 0.986 | 0.008 | 0.006 | 1 |
| H858 | Uganda | Tororo | TR | *Tbb* | - | Cattle | 1988 | 0.973 | 0.009 | 0.018 | 1 |
| H862 | Uganda | Tororo | TR | *Tbb* | - | Cattle | 1988 | 0.977 | 0.007 | 0.016 | 1 |
| H578 | Uganda | Tororo | TR | *Tbb* | - | Cattle | 1991 | 0.399 | 0.003 | 0.598 | 3 |
| H582 | Uganda | Tororo | TR | *Tbb* | - | Cattle | 2005 | 0.916 | 0.007 | 0.078 | 1 |
| H285 | Uganda | Tororo | TR | *Tbb* | - | Hippo | 1961 | 0.995 | 0.002 | 0.003 | 1 |
| H596 | Uganda | Tororo | TR | *Tbr* | ₊ | Human | 1990 | 0.990 | 0.003 | 0.007 | 1 |
| H601 | Uganda | Tororo | TR | *Tbr* | ₊ | Human | 1990 | 0.028 | 0.003 | 0.969 | 3 |
| H605 | Uganda | Tororo | TR | *Tbr* | ₊ | Human | 1990 | 0.003 | 0.003 | 0.994 | 3 |
| H617 | Uganda | Tororo | TR | *Tbr* | ₊ | Human | 1990 | 0.003 | 0.003 | 0.994 | 3 |
| H618 | Uganda | Tororo | TR | *Tbr* | ₊ | Human | 1990 | 0.004 | 0.008 | 0.988 | 1 |
| H620 | Uganda | Tororo | TR | *Tbr* | ₊ | Human | 1990 | 0.004 | 0.005 | 0.991 | 1 |
| H840 | Uganda | Tororo | TR | *Tbr* | ₊ | Human | 1990 | 0.009 | 0.007 | 0.984 | 1 |
| H847 | Uganda | Tororo | TR | *Tbr* | ₊ | Human | 1990 | 0.014 | 0.006 | 0.980 | 3 |
| H866 | Uganda | Tororo | TR | *Tbr* | ₊ | Human | 1990 | 0.005 | 0.003 | 0.992 | 1 |
| H868 | Uganda | Tororo | TR | *Tbr* | ₊ | Human | 1990 | 0.035 | 0.011 | 0.954 | 1 |
| H579 | Uganda | Tororo | TR | *Tbr* | ₊ | Human | 1991 | 0.382 | 0.007 | 0.611 | 3 |
| H601 | Uganda | Tororo | TR | *Tbr* | ₊ | Human | 1991 | 0.028 | 0.003 | 0.969 | 3 |
| H607 | Uganda | Tororo | TR | *Tbr* | ₊ | Human | 1991 | 0.978 | 0.005 | 0.017 | 1 |
| H612 | Uganda | Tororo | TR | *Tbr* | ₊ | Human | 1991 | 0.010 | 0.050 | 0.940 | 3 |
| H838 | Uganda | Tororo | TR | *Tbr* | ₊ | Human | 1991 | 0.619 | 0.003 | 0.378 | 1 |
| H838_1 | Uganda | Tororo | TR | *Tbr* | ₊ | Human | 1991 | 0.011 | 0.006 | 0.983 | 3 |
| H850 | Uganda | Tororo | TR | *Tbr* | ₊ | Human | 1992 | 0.004 | 0.006 | 0.990 | 3 |
| Try056 | Kenya | Tororo | TR | *Tbr* | ₊ | Human | 1992 | 0.003 | 0.993 | 0.004 | 2 |
| H855 | Uganda | Tororo | TR | *Tbr* | ₊ | Human | 1988 | 0.005 | 0.007 | 0.988 | 3 |
| H856 | Uganda | Tororo | TR | *Tbr* | ₊ | Human |  | 0.003 | 0.004 | 0.993 | 3 |
| H859 | Uganda | Tororo | TR | *Tbr* | ₊ | Human |  | 0.004 | 0.004 | 0.992 | 3 |
| H860 | Uganda | Tororo | TR | *Tbr* | ₊ | Human |  | 0.023 | 0.018 | 0.960 | 3 |
| H861 | Uganda | Tororo | TR | *Tbr* | ₊ | Human |  | 0.004 | 0.005 | 0.991 | 3 |
| Try046 | Kenya | Tororo | TR | *Tbr* | ₊ | Tsetse fly | 1960 | 0.003 | 0.994 | 0.003 | 2 |
| Try053 | Kenya | Tororo | TR | *Tbr* | ₊ | Tsetse fly | 1960 | 0.002 | 0.995 | 0.003 | 2 |
| Try001 | Kenya | Teso | TS | *Tbr* | ₊ | Human | 2009 | 0.002 | 0.995 | 0.003 | 2 |
