# Supplementary material for: Genetic Diversity and Population Structure of Trypanosoma brucei in Uganda: Implications for the Epidemiology of Sleeping Sickness and Nagana
Source: PLoS Negl Trop Dis. 2015 Feb 19;9(2):e0003353. doi: 10.1371/journal.pntd.0003353 (PMC4335064; doi:10.1371/journal.pntd.0003353)
Supplement: S2 Table — The first two columns report the locus name. The next two columns show the DNA sequence of the forward and reverse primers, specifying in parenthesis the type of fluorescent dye used for each one. The next two columns list the repeat motif for each locus and the range of length of the alleles in base pairs (bp). The second to the last column reports the chromosomal location of each locus according to the reference in the last column. (DOCX) [file pntd.0003353.s002.docx]

| Locus | Forward Primer | Reverse Primer | Motif | Size range (bp) | Chrom. Loc. | Source |
| --- | --- | --- | --- | --- | --- | --- |
| Tryp51 | [FAM]-TGACCCGTGAGAAGTGAAC | GCGCATCTACAGGCATAGAC | (ATT) | 187-238 | 9 | Sistrom *et al* 2012 |
| Tryp52 | [ALEXA 532]-GCATCATTGACGTCGACCC | TAACAACCACTGGGACCGC | (GT) | 201-231 | 11 | Sistrom *et al* 2012 |
| Tryp53 | [ALEXA 546]-GTACAGCCACGTGCAAACC | TGTACACAATCGGGTGGATG | (AC) | 200-254 | 7 | Sistrom *et al* 2012 |
| Tryp54 | [ROX]-AGTCGGCGTGATGGTACTC | TTCAGCCCACAAACAACCG | (AAAT) | 144-176 | 10 | Sistrom *et al* 2012 |
| Tryp55 | [FAM]-AATTCAACCCCAACAGCCC | CTCGTTCAATGACTTGCCCC | (GT) | 208-246 | 5 | Sistrom *et al* 2012 |
| Tryp59 | [ALEXA 532]-GAGGCAATCGCAGTGTGTG | CGCACGTTTCACCATCCTC | (GT) | 209-225 | 9 | Sistrom *et al* 2012 |
| Tryp61 | [ALEXA546]-ACTCGCGACAGACCATGAG | ACAGGAGAGTGTTGTGAGTG | (ATT) | 179-215 | 11 | Sistrom *et al* 2012 |
| Tryp62 | [ROX]-AAGGCGACCAACTTCAACC | GTTGTCATCGGCTTGCTCC | (AC) | 153-177 | 11 | Sistrom *et al* 2012 |
| Tryp65 | [ALEXA 546]-GGAGGTAAACTTGATTCGGGTG | ACGACAACAGCGACAAAGC | (ATT) | 207-234 | 9 | Sistrom *et al* 2012 |
| Tryp66 | [ROX]-TCCTCGTACCTTTTCTCTCAC | ACGAAATTTAGGTGTGAAAGCTG | (ATT) | 384-396 | 5 | Sistrom *et al* 2012 |
| Tryp67 | [FAM]-GTTGCTGAGGTGCAACTGG | GTCGTCAGGCACCAAAACG | (GTT) | 151-178 | 7 | Sistrom *et al* 2012 |
| TB1/8 | [FAM]-AGGTTTAGTGCATGTCGGA | CCTGTTGTACGGAGGTCA | (CA) | 97-117 | 1 | Balmer *et al* 2006 |
| TB5/2 | [HEX]-CAACCGAAAGTAAGGGGAAC | TCTCGCCTTCTTTGCCC | (AT) | 83-107 | 5 | Balmer *et al* 2006 |
| TB6/7 | [HEX]-AAGCTGACAGGTGGTTGA | GAACATGCGTGCGTGTG | (AT) | 104-136 | 6 | Balmer *et al* 2006 |
| TB9/6 | [HEX]-TGATTCATTGGTTAAGACAGG | AATGATAACTGCGGATTACAC | (AC) | 124-158 | 9 | Balmer *et al* 2006 |
| TB10/5 | [FAM]-AAAGGCGATATGTTATTATTGA | ATTGGGTATACTGTCCCTCA | (TA) | 79-115 | 10 | Balmer *et al* 2006 |
| TB11/13 | [FAM]-CAAGAACTCTGCATTGAGC | ATCTGTTGGCGATGGTGA | (AT) | 125-161 | 11 | Balmer *et al* 2006 |
