# Supplementary material for: Genetic Diversity and Population Structure of Trypanosoma brucei in Uganda: Implications for the Epidemiology of Sleeping Sickness and Nagana
Source: PLoS Negl Trop Dis. 2015 Feb 19;9(2):e0003353. doi: 10.1371/journal.pntd.0003353 (PMC4335064; doi:10.1371/journal.pntd.0003353)
Supplement: S3 Table — The first three columns list the geographic origin (Origin), the district abbreviations (Code), and the host (Host). The forth column shows the number of strains for each host (N). The following three columns provide information on the Trypanosoma species infection in the samples other than T. brucei (T. vivax = Tv; T, congolense) and the occurrence of mixed Tv and Tc infections. The next two columns summarize the number of Tbb and Tbr samples, according to the SRA test. The final column reports the number of samples that did not produce PCR products, likely due to low DNA concentration and/or poor quality. (DOCX) [file pntd.0003353.s003.docx]

| **Origin** | **Code** | **Host** | **N** | ***Tv*** | ***Tc*** | ***Tv* & *Tc*** | ***Tbb*** | ***Tbr*** | **No amplification** |
| --- | --- | --- | --- | --- | --- | --- | --- | --- | --- |
|  |  |  |  |  |  |  |  |  |  |
|  |  |  |  |  |  |  |  |  |  |
| Amuru | AM | Cattle | 46 | 45 | 1 | 0 | 0 | 0 | 0 |
|  |  | Goats | 2 | 2 | 0 | 0 | 0 | 0 | 0 |
| Bugiri | BG | Pig | 2 | 0 | 0 | 0 | 2 | 0 | 0 |
| Busia | BU | Cattle | 11 | 3 | 1 | 0 | 1 | 2 | 4 |
|  |  | Pig | 1 | 0 | 0 | 0 | 1 | 0 | 0 |
| Busoga | BS | Tsetse | 1 | 0 | 0 | 0 | 0 | 1 | 0 |
|  |  | Dog | 1 | 0 | 0 | 0 | 0 | 1 | 0 |
| Central Nyanza | CN | Cattle | 4 | 0 | 0 | 0 | 0 | 4 | 0 |
|  |  | Tsetse | 1 | 0 | 0 | 0 | 0 | 1 | 0 |
|  |  | Wildlife | 2 | 0 | 0 | 0 | 0 | 2 | 0 |
| Dokolo | DK | Cattle | 33 | 28 | 1 | 0 | 1 | 0 | 3 |
| Kaberamaido | KA | Cattle | 21 | 13 | 1 | 5 | 2 | 0 | 0 |
| Kayunga | KY | Pig | 2 | 0 | 0 | 0 | 2 | 0 | 0 |
| Kole | KO | Cattle | 101 | 51 | 5 | 2 | 20 | 5 | 18 |
| Lira | LR | Cattle | 3 | 2 | 1 | 0 | 0 | 0 | 0 |
| Mukono | MK | Cattle | 1 | 0 | 0 | 0 | 1 | 0 | 0 |
|  |  | Pig | 5 | 0 | 0 | 0 | 5 | 0 | 0 |
| Sidende | SD | Tsetse | 1 | 0 | 0 | 0 | 0 | 1 | 0 |
| South Nyanza | SN | Cattle | 2 | 0 | 0 | 0 | 0 | 2 | 0 |
|  |  | Sheep | 1 | 0 | 0 | 0 | 0 | 1 | 0 |
|  |  | Wildlife | 2 | 0 | 0 | 0 | 0 | 2 | 0 |
|  |  | Tsetse | 4 | 0 | 0 | 0 | 0 | 4 | 0 |
| Soroti | SRT | Cattle | 15 | 1 | 0 | 0 | 10 | 3 | 1 |
| Tororo | TR | Cattle | 5 | 0 | 1 | 0 | 2 | 0 | 2 |
|  |  | Tsetse | 2 | 0 | 0 | 0 | 0 | 2 | 0 |
| **Total** |  |  | **269** | **145** | **11** | **7** | **49** | **31** | **28** |
