# Supplementary material for: Genetic Diversity and Population Structure of Trypanosoma brucei in Uganda: Implications for the Epidemiology of Sleeping Sickness and Nagana
Source: PLoS Negl Trop Dis. 2015 Feb 19;9(2):e0003353. doi: 10.1371/journal.pntd.0003353 (PMC4335064; doi:10.1371/journal.pntd.0003353)
Supplement: S4 Table — P-values and their Standard Errors (S.E>) are reported in the last column (DOCX) [file pntd.0003353.s004.docx]

| Locus#1 | Locus#2 | P-Value | S.E. |
| --- | --- | --- | --- |
| Locus_51 | Locus_52 | 0.000 | 0.000 |
| Locus_51 | Locus_53 | 0.032 | 0.016 |
| Locus_52 | Locus_53 | 0.000 | 0.000 |
| Locus_51 | Locus_54 | 0.000 | 0.000 |
| Locus_52 | Locus_54 | 0.000 | 0.000 |
| Locus_53 | Locus_54 | 0.000 | 0.000 |
| Locus_51 | Locus_55 | 0.000 | 0.000 |
| Locus_52 | Locus_55 | 0.000 | 0.000 |
| Locus_53 | Locus_55 | 0.024 | 0.014 |
| Locus_54 | Locus_55 | 0.000 | 0.000 |
| Locus_51 | Locus_59 | 0.000 | 0.000 |
| Locus_52 | Locus_59 | 0.000 | 0.000 |
| Locus_53 | Locus_59 | 0.000 | 0.000 |
| Locus_54 | Locus_59 | 0.000 | 0.000 |
| Locus_55 | Locus_59 | 0.000 | 0.000 |
| Locus_51 | Locus_61 | 0.083 | 0.024 |
| Locus_52 | Locus_61 | 0.000 | 0.000 |
| Locus_53 | Locus_61 | 0.042 | 0.017 |
| Locus_54 | Locus_61 | 0.000 | 0.000 |
| Locus_55 | Locus_61 | 0.025 | 0.012 |
| Locus_59 | Locus_61 | 0.003 | 0.002 |
| Locus_51 | Locus_62 | 0.004 | 0.004 |
| Locus_52 | Locus_62 | 0.000 | 0.000 |
| Locus_53 | Locus_62 | 0.000 | 0.000 |
| Locus_54 | Locus_62 | 0.000 | 0.000 |
| Locus_55 | Locus_62 | 0.000 | 0.000 |
| Locus_59 | Locus_62 | 0.000 | 0.000 |
| Locus_61 | Locus_62 | 0.000 | 0.000 |
| Locus_51 | Locus_65 | 0.000 | 0.000 |
| Locus_52 | Locus_65 | 0.000 | 0.000 |
| Locus_53 | Locus_65 | 0.000 | 0.000 |
| Locus_54 | Locus_65 | 0.000 | 0.000 |
| Locus_55 | Locus_65 | 0.003 | 0.003 |
| Locus_59 | Locus_65 | 0.000 | 0.000 |
| Locus_61 | Locus_65 | 0.022 | 0.009 |
| Locus_62 | Locus_65 | 0.000 | 0.000 |
| Locus_51 | Locus_66 | 0.068 | 0.016 |
| Locus_52 | Locus_66 | 0.000 | 0.000 |
| Locus_53 | Locus_66 | 0.000 | 0.000 |
| Locus_54 | Locus_66 | 0.000 | 0.000 |
| Locus_55 | Locus_66 | 0.000 | 0.000 |
| Locus_59 | Locus_66 | 0.225 | 0.019 |
| Locus_61 | Locus_66 | 0.000 | 0.000 |
| Locus_62 | Locus_66 | 0.000 | 0.000 |
| Locus_65 | Locus_66 | 0.018 | 0.006 |
| Locus_51 | Locus_67 | 0.000 | 0.000 |
| Locus_52 | Locus_67 | 0.000 | 0.000 |
| Locus_53 | Locus_67 | 0.000 | 0.000 |
| Locus_54 | Locus_67 | 0.000 | 0.000 |
| Locus_55 | Locus_67 | 0.000 | 0.000 |
| Locus_59 | Locus_67 | 0.000 | 0.000 |
| Locus_61 | Locus_67 | 0.010 | 0.008 |
| Locus_62 | Locus_67 | 0.000 | 0.000 |
| Locus_65 | Locus_67 | 0.000 | 0.000 |
| Locus_66 | Locus_67 | 0.003 | 0.002 |
| Locus_51 | Tb5_2 | 0.000 | 0.000 |
| Locus_52 | Tb5_2 | 0.000 | 0.000 |
| Locus_53 | Tb5_2 | 0.000 | 0.000 |
| Locus_54 | Tb5_2 | 0.000 | 0.000 |
| Locus_55 | Tb5_2 | 0.000 | 0.000 |
| Locus_59 | Tb5_2 | 0.000 | 0.000 |
| Locus_61 | Tb5_2 | 0.521 | 0.032 |
| Locus_62 | Tb5_2 | 0.000 | 0.000 |
| Locus_65 | Tb5_2 | 0.000 | 0.000 |
| Locus_66 | Tb5_2 | 0.018 | 0.009 |
| Locus_67 | Tb5_2 | 0.000 | 0.000 |
| Locus_51 | Tb6_7 | 0.000 | 0.000 |
| Locus_52 | Tb6_7 | 0.000 | 0.000 |
| Locus_53 | Tb6_7 | 0.000 | 0.000 |
| Locus_54 | Tb6_7 | 0.000 | 0.000 |
| Locus_55 | Tb6_7 | 0.000 | 0.000 |
| Locus_59 | Tb6_7 | 0.000 | 0.000 |
| Locus_61 | Tb6_7 | 0.000 | 0.000 |
| Locus_62 | Tb6_7 | 0.000 | 0.000 |
| Locus_65 | Tb6_7 | 0.000 | 0.000 |
| Locus_66 | Tb6_7 | 0.000 | 0.000 |
| Locus_67 | Tb6_7 | 0.000 | 0.000 |
| Tb5_2 | Tb6_7 | 0.000 | 0.000 |
| Locus_51 | Tb9_6 | 0.000 | 0.000 |
| Locus_52 | Tb9_6 | 0.000 | 0.000 |
| Locus_53 | Tb9_6 | 0.000 | 0.000 |
| Locus_54 | Tb9_6 | 0.000 | 0.000 |
| Locus_55 | Tb9_6 | 0.000 | 0.000 |
| Locus_59 | Tb9_6 | 0.000 | 0.000 |
| Locus_61 | Tb9_6 | 0.000 | 0.000 |
| Locus_62 | Tb9_6 | 0.000 | 0.000 |
| Locus_65 | Tb9_6 | 0.000 | 0.000 |
| Locus_66 | Tb9_6 | 0.000 | 0.000 |
| Locus_67 | Tb9_6 | 0.000 | 0.000 |
| Tb5_2 | Tb9_6 | 0.000 | 0.000 |
| Tb6_7 | Tb9_6 | 0.000 | 0.000 |
| Locus_51 | Tb10_5 | 0.010 | 0.008 |
| Locus_52 | Tb10_5 | 0.000 | 0.000 |
| Locus_53 | Tb10_5 | 0.000 | 0.000 |
| Locus_54 | Tb10_5 | 0.000 | 0.000 |
| Locus_55 | Tb10_5 | 0.000 | 0.000 |
| Locus_59 | Tb10_5 | 0.000 | 0.000 |
| Locus_61 | Tb10_5 | 0.000 | 0.000 |
| Locus_62 | Tb10_5 | 0.000 | 0.000 |
| Locus_65 | Tb10_5 | 0.000 | 0.000 |
| Locus_66 | Tb10_5 | 0.000 | 0.000 |
| Locus_67 | Tb10_5 | 0.000 | 0.000 |
| Tb5_2 | Tb10_5 | 0.000 | 0.000 |
| Tb6_7 | Tb10_5 | 0.000 | 0.000 |
| Tb9_6 | Tb10_5 | 0.000 | 0.000 |
| Locus_51 | Tb1-8 | 0.000 | 0.000 |
| Locus_52 | Tb1-8 | 0.000 | 0.000 |
| Locus_53 | Tb1-8 | 0.000 | 0.000 |
| Locus_54 | Tb1-8 | 0.000 | 0.000 |
| Locus_55 | Tb1-8 | 0.000 | 0.000 |
| Locus_59 | Tb1-8 | 0.000 | 0.000 |
| Locus_61 | Tb1-8 | 0.000 | 0.000 |
| Locus_62 | Tb1-8 | 0.000 | 0.000 |
| Locus_65 | Tb1-8 | 0.000 | 0.000 |
| Locus_66 | Tb1-8 | 0.000 | 0.000 |
| Locus_67 | Tb1-8 | 0.000 | 0.000 |
| Tb5_2 | Tb1-8 | 0.000 | 0.000 |
| Tb6_7 | Tb1-8 | 0.000 | 0.000 |
| Tb9_6 | Tb1-8 | 0.000 | 0.000 |
| Tb10_5 | Tb1-8 | 0.000 | 0.000 |
| Locus_51 | Tb11-13 | 0.000 | 0.000 |
| Locus_52 | Tb11-13 | 0.000 | 0.000 |
| Locus_53 | Tb11-13 | 0.000 | 0.000 |
| Locus_54 | Tb11-13 | 0.000 | 0.000 |
| Locus_55 | Tb11-13 | 0.000 | 0.000 |
| Locus_59 | Tb11-13 | 0.000 | 0.000 |
| Locus_61 | Tb11-13 | 0.000 | 0.000 |
| Locus_62 | Tb11-13 | 0.000 | 0.000 |
| Locus_65 | Tb11-13 | 0.000 | 0.000 |
| Locus_66 | Tb11-13 | 0.000 | 0.000 |
| Locus_67 | Tb11-13 | 0.000 | 0.000 |
| Tb5_2 | Tb11-13 | 0.000 | 0.000 |
| Tb6_7 | Tb11-13 | 0.000 | 0.000 |
| Tb9_6 | Tb11-13 | 0.000 | 0.000 |
| Tb10_5 | Tb11-13 | 0.000 | 0.000 |
| Tb1-8 | Tb11-13 | 0.000 | 0.000 |
