# Supplementary material for: Genetic Diversity and Population Structure of Trypanosoma brucei in Uganda: Implications for the Epidemiology of Sleeping Sickness and Nagana
Source: PLoS Negl Trop Dis. 2015 Feb 19;9(2):e0003353. doi: 10.1371/journal.pntd.0003353 (PMC4335064; doi:10.1371/journal.pntd.0003353)
Supplement: S5 Table — Asterisks denote statistically significant values (*P<0.05; **P<0.01). (DOCX) [file pntd.0003353.s005.docx]

|  | AP | BG | BU | BS | DK | KA | KP | KY | KB | KO | LR | MK | | PL | | SR | | TR | | TS | | CN | | SN | | SD | |  | |
| --- | --- | --- | --- | --- | --- | --- | --- | --- | --- | --- | --- | --- | --- | --- | --- | --- | --- | --- | --- | --- | --- | --- | --- | --- | --- | --- | --- | --- | --- |
| AP | 0.00 |  |  |  |  |  |  |  |  |  |  |  | |  | |  | |  | |  | |  | |  | |  | |  | |
| BG | -0.10 | 0.00 |  |  |  |  |  |  |  |  |  |  | |  | |  | |  | |  | |  | |  | |  | |  | |
| BU | 0.16 | 0.13 | 0.00 |  |  |  |  |  |  |  |  |  | |  | |  | |  | |  | |  | |  | |  | |  | |
| BS | -0.02 | 0.03 | 0.05** | 0.00 |  |  |  |  |  |  |  |  | |  | |  | |  | |  | |  | |  | |  | |  | |
| DK | 0.16 | 0.05 | 0.09** | 0.01 | 0.00 |  |  |  |  |  |  |  | |  | |  | |  | |  | |  | |  | |  | |  | |
| KA | 0.17 | 0.14** | 0.2** | 0.1** | 0.03 | 0.00 |  |  |  |  |  |  | |  | |  | |  | |  | |  | |  | |  | |  | |
| KP | 0.38 | -0.82 | -0.97 | -0.79 | -0.15 | -0.23 | 0.00 |  |  |  |  |  | |  | |  | |  | |  | |  | |  | |  | |  | |
| KY | -0.06 | 0.05 | 0.14* | 0.03 | 0.18 | 0.33* | -0.89 | 0.00 |  |  |  |  | |  | |  | |  | |  | |  | |  | |  | |  | |
| KB | 0.00 | -0.11 | 0.05 | -0.11 | -0.04 | -0.34 | -0.50 | -0.06 | 0.00 |  |  |  | |  | |  | |  | |  | |  | |  | |  | |  | |
| KO | 0.32 | 0.01 | 0.13** | 0.01 | 0.23** | 0.26** | -0.10 | -0.02 | 0.19 | 0.00 |  |  | |  | |  | |  | |  | |  | |  | |  | |  | |
| LR | -0.01 | -0.03 | 0.12** | 0.00 | -0.01 | 0.02 | -0.35 | 0.07 | -0.33 | 0.18** | 0.00 |  | |  | |  | |  | |  | |  | |  | |  | |  | |
| MK | 0.19 | 0.04 | 0.27** | 0.16** | 0.35** | 0.48** | -0.50 | 0.06 | 0.19 | 0.25** | 0.22* | 0.00 | |  | |  | |  | |  | |  | |  | |  | |  | |
| PL | 0.11 | 0.24** | 0.24** | 0.15** | 0.06** | 0.03* | -0.50 | 0.37** | -0.51 | 0.26** | 0.05 | 0.49** | | 0.00 | |  | |  | |  | |  | |  | |  | |  | |
| SR | 0.02 | 0.08* | 0.18** | 0.07** | -0.03 | 0.01 | -0.60 | 0.13 | -0.21 | 0.12** | -0.04 | 0.32** | | 0.09** | | 0.00 | |  | |  | |  | |  | |  | |  | |
| TR | -0.08 | 0.05** | 0.05** | 0.01 | -0.01 | 0.07** | -0.78 | 0.08 | -0.14 | 0.01* | 0.01 | 0.2** | | 0.15** | | 0.06** | | 0.00 | |  | |  | |  | |  | |  | |
| TS | 0.44 | 0.24 | 0.14 | 0.17 | 0.47 | 0.52 | -0.43 | 0.11 | 0.26 | 0.44 | 0.38 | 0.27 | | 0.48 | | 0.37 | | 0.23 | | 0.00 | |  | |  | |  | |  | |
| CN | 0.33 | 0.23** | 0.05* | 0.16** | 0.3** | 0.42** | -1.07 | 0.2* | 0.22 | 0.31** | 0.29** | 0.27 | | 0.39** | | 0.31** | | 0.18** | | -0.05 | | 0.00 | |  | |  | |  | |
| SN | 0.40 | 0.25** | 0.08** | 0.16** | 0.31** | 0.43** | -0.75 | 0.21** | 0.26 | 0.28** | 0.29** | 0.31** | | 0.41** | | 0.32** | | 0.19** | | -0.02 | | -0.05 | | 0.00 | |  | |  | |
| SD | 0.33 | -0.12 | -0.06 | -0.12 | 0.10 | 0.22 | -0.75 | -0.39 | -0.25 | 0.08 | 0.10 | -0.06 | | 0.21 | | 0.05 | | -0.06 | | -0.29 | | -0.26 | | -0.02 | | 0.00 | |  | |
|  |  |  |  |  |  |  |  |  |  |  |  |  |  |  |  |  |  |  |  |  |  |  |  |  |  |  |  |  |  |
|  |  |  |  |  |  |  |  |  |  |  |  |  |  |  |  |  |  |  |  |  |  |  |  |  |  |  |  |  |  |
|  |  |  |  |  |  |  |  |  |  |  |  |  |  |  |  |  |  |  |  |  |  |  |  |  |  |  |  |  |  |
|  |  |  |  |  |  |  |  |  |  |  |  |  |  |  |  |  |  |  |  |  |  |  |  |  |  |  |  |  |  |
|  |  |  |  |  |  |  |  |  |  |  |  |  |  |  |  |  |  |  |  |  |  |  |  |  |  |  |  |  |  |
