# Supplementary material for: Genetic Diversity and Population Structure of Trypanosoma brucei in Uganda: Implications for the Epidemiology of Sleeping Sickness and Nagana
Source: PLoS Negl Trop Dis. 2015 Feb 19;9(2):e0003353. doi: 10.1371/journal.pntd.0003353 (PMC4335064; doi:10.1371/journal.pntd.0003353)
Supplement: S6 Table — Two asterisks indicate significance at P<0.01. (DOCX) [file pntd.0003353.s006.docx]

| Cluster | 1 | 2 | 3 |
| --- | --- | --- | --- |
| 1 | 0 |  |  |
| 2 | 0.16** | 0 |  |
| 3 | 0.21** | 0.27** | 0 |
