# Supplementary figures and images for: Genetic Diversity and Population Structure of Trypanosoma brucei in Uganda: Implications for the Epidemiology of Sleeping Sickness and Nagana
Source: PLoS Negl Trop Dis. 2015 Feb 19;9(2):e0003353. doi: 10.1371/journal.pntd.0003353 (PMC4335064; doi:10.1371/journal.pntd.0003353)

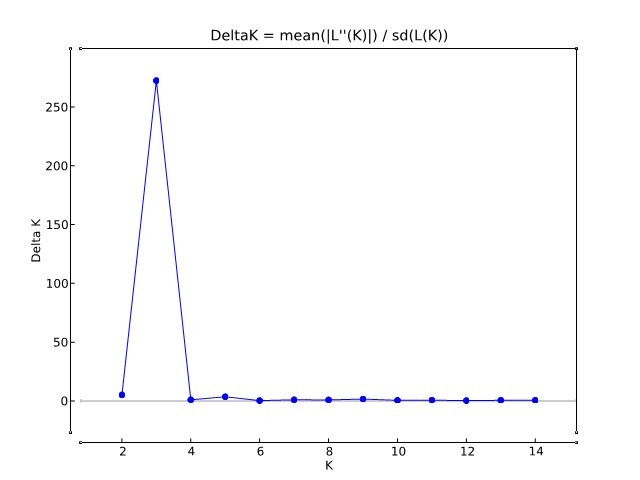

Supplement: S1 Fig — The highest peak at ΔK represents the most appropriate number of genetic clusters (K = 3). (JPG) [file pntd.0003353.s007.jpg]

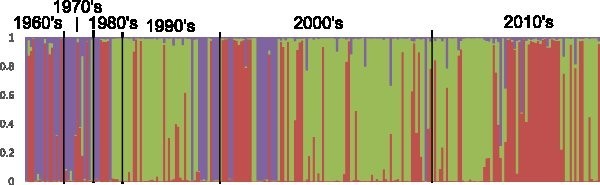

Supplement: S2 Fig — Dark bold vertical lines group the samples by decade of collections from 1970’s to 2010’s. Numbers on the vertical axis (0–1) refer to the individual assignment of each sample the three genetic clusters (red, purple, and green). (JPG) [file pntd.0003353.s008.jpg]

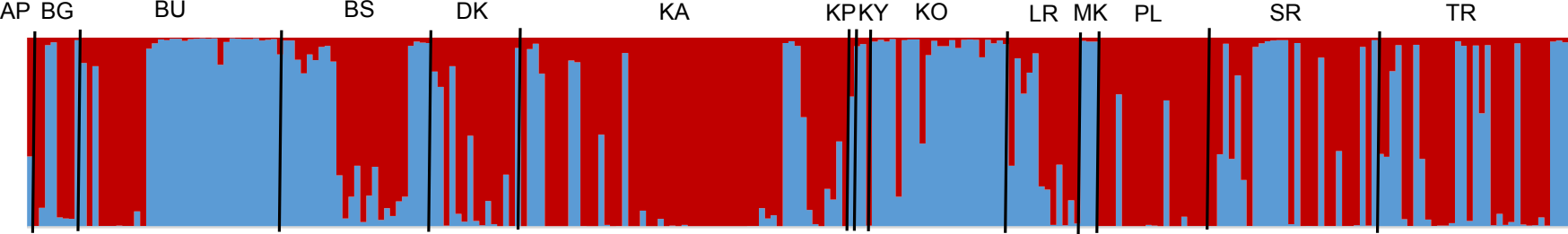

Supplement: S3 Fig — Dark bold vertical lines group the samples by district (symbol for each district is listed above the plot. The most likely number of cluster is 2 (K = 2). The vertical axis represents the individual assignment of each sample the two inferred clusters (red, blue). (PDF) [file pntd.0003353.s009.pdf]
